# Effective mobilities for thin-film flows on micropillar arrays

**DOI:** 10.1140/epje/s10189-026-00615-6

**Published:** 2026-07-31

**Authors:** Raphael Saiseau, Stefan Karpitschka

**Affiliations:** Department of Physics, University of Konstanz, 78457 Konstanz, Germany

## Abstract

**Abstract:**

Micropillar-textured surfaces provide versatile platforms for controlling wetting, imbibition, and droplet dynamics, and also serve as model systems for engineered textured surfaces used in applications ranging from thermal management and microfluidics to semiconductor processing. While spontaneous hemiwicking in such geometries has been extensively studied, a unified framework for thin-film modeling that captures both capillary-driven and Marangoni-driven transport across arbitrary film thicknesses has remained lacking. Here, we derive explicit analytical mobility expressions for lubrication-based modeling of wetting dynamics on periodic micropillar arrays. Using an equivalent microchannel formulation and a Fourier series solution of the Stokes equations, we obtain exact mobilities for both pressure-driven and Marangoni-driven flows, valid for film thicknesses both below and above the pillar height. The resulting formulation captures the interplay between film thickness, pillar height, and the lateral confinement scale, and connects confined transport, overtopped films, and effective slip behavior within a single framework. In particular, Marangoni-driven transport exhibits a stronger saturation with increasing pillar height than pressure-driven transport once lateral confinement dominates. These mobility laws recover known hemiwicking limits and provide the constitutive ingredient needed to extend lubrication theory to complex wetting dynamics on textured substrates.

**Graphical abstract:**

**Figure:**
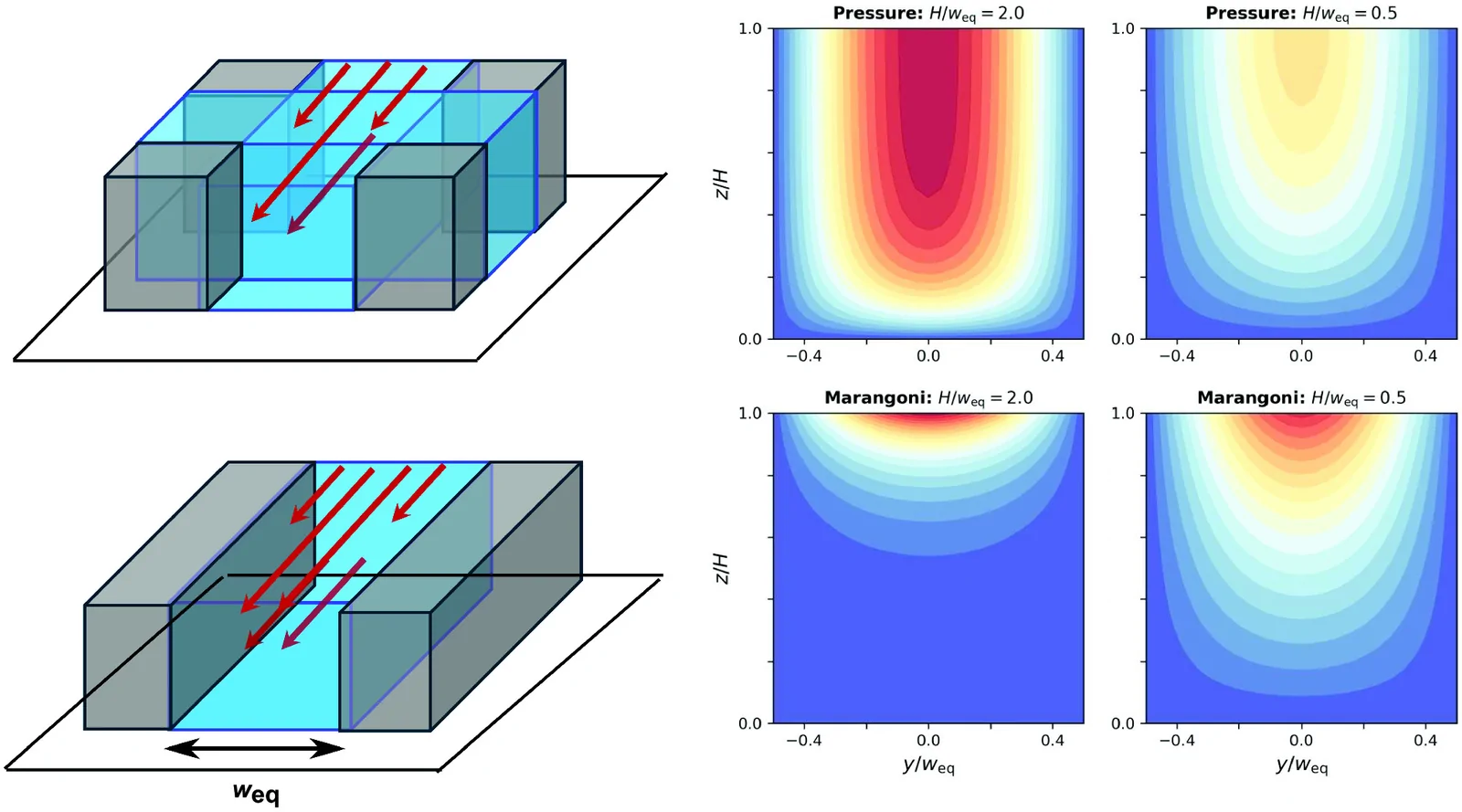

**Supplementary Information:**

The online version contains supplementary material available at 10.1140/epje/s10189-026-00615-6.

## Introduction

Dynamic wetting on adaptive, flexible, and switchable substrates is a setting in which the wetting response is not fixed by a rigid, inert surface but evolves together with the liquid through a strong coupling between the (de)wetting hydrodynamics and the state of the substrate [1]. Such coupling generically introduces additional length and time scales and reshapes the dissipation: the deformation near the contact line for soft and elastic substrates [2–4], the swelling and reorganization for adaptive layers such as polymer brushes and gels [5], or the transport through a thin mobile sublayer on lubricant-infused surfaces [6]. A micropillar array is a particularly controlled, perhaps simplest, member of this family. The same texture switches its effective wetting state, from pinned or hemiwicking to a lubricated, effectively slippery film once the liquid has overtopped the pillars. Depending on pillar geometry, spacing, and surface chemistry, such textures support a broad spectrum of wetting states, from superhydrophobicity to complete imbibition [7–9].

Due to their geometric control over wetting behavior and their ease of fabrication using standard lithographic methods [7, 10], micropillar arrays constitute model systems for studying liquid transport through textured surfaces. This process is central to a broad range of natural and technological processes, including capillary wicking, microfluidic transport, coating of structured substrates, and wet processing in microelectronics [7, 11]. Thus, they provide an intermediate, model step to isolate the hydrodynamics of a permeable, dissipative surface layer, before going toward soft and permeable adaptive substrates such as polymer brushes, which may be viewed as a soft, molecular-scale analog of a pillar forest.

Hemiwicking, where liquid spontaneously invades the texture while maintaining a free interface near the pillar tops, is an especially important regime, both for applications and for fundamental studies of capillary transport [10, 12]. Its geometry can be tuned through the pillar pattern, enabling functionalities such as directed spreading, deposit shaping, and liquid rectification [9, 13–16]. It is also relevant to passive cooling, inkjet printing, microfluidic actuation, and slippery liquid-infused porous surfaces (SLIPS) [6, 16–18].

Classical descriptions of hemiwicking rely on geometric or energetic criteria to determine whether spontaneous imbibition occurs [7, 9, 10, 19]. In this picture, a capillary driving force, localized at the imbibition front, is balanced by viscous dissipation distributed through the already wetted textured region [8, 12], leading to a Washburn-like diffusive law for the imbibed length $L(t)\sim \sqrt{D_w t}$ [8, 12, 20, 21]. Significant effort has therefore been devoted to predicting the wicking coefficient $D_w$ analytically [8, 10, 12, 22–25]. Among these approaches, several works use simplified channel-like flow paths to replace the detailed textured geometry (hydraulic diameter approximations or equivalent open-channel constructions [25–27]), capturing the role of lateral confinement in the viscous resistance to wicking.

A common limitation of these theories, however, is that they assume a constant film thickness equal to the pillar height, h=H. This approximation is appropriate for purely capillary-driven imbibition of totally wetting liquids, but it becomes insufficient as soon as additional stresses develop along the imbibed film and generate thickness variations. This is the case, for example, when surface tension gradients induce Marangoni stresses [28], or when evaporation and condensation act as local volumetric sources and sinks [29]. More generally, the classical picture does not directly account for situations in which a droplet and an imbibed film coexist and evolve together, or where the liquid overtops the texture, h>H.

To describe such situations, a useful route is to seek an effective long-wave description of the mean flow on lateral scales much larger than the texture periodicity. In that setting, the micropillar array is not resolved in full detail, but is described as an effective medium to derive transport coefficients that account for its influence on the averaged motion. Within this effective description, the local film thickness h(x,y,t) evolves according to the thin-film equation [30, 31]

1 $$\begin{aligned} \frac{\partial h}{\partial t} + \nabla \cdot \textbf{q} = 0, \end{aligned}$$

describing mass conservation integrated over the film thickness. In Stokes flow, the flux $\textbf{q}$ couples linearly to driving forces through hydrodynamic mobility tensors $\textbf{M}(h)$ and $\textbf{N}(h)$, symmetric and positive-definite matrices that encapsulate the geometry of the system, boundary conditions, and fluid viscosity:

2 $$\begin{aligned} \textbf{q} = -\textbf{M}(h)\nabla p + \textbf{N}(h)\nabla \gamma . \end{aligned}$$

where *p* denotes the generalized pressure potential (including capillary pressure, disjoining pressure, and gravity) and γ the surface tension. Thus, $\textbf{M}(h)$ and $\textbf{N}(h)$ represent the mobilities with respect to pressure- and surface-driven flow, respectively.

In a one-dimensional/unit cell formulation, which we explore here, these mobilities reduce to scalar functions M(h) and N(h). For flat substrates, classical lubrication theory gives $M(h)=h^3/(3\eta )$ for pressure-driven flow and $N(h)=h^2/(2\eta )$ for Marangoni-driven flow, with η the viscosity [30]. Their counterparts for flows inside micropillar arrays, however, have not been established in a general form valid across arbitrary film thicknesses. This effective approach is appropriate only as long as one is interested in mean flow properties on scales well above the texture pitch, and is also consistent with descriptions of effective slip boundary conditions of flows over patterned or topographically structured surfaces [32]: Local recirculations, detailed meniscus shapes, complex boundary geometries, and other flow structures on the scale of a single unit cell are not resolved.

**Fig. 1:**
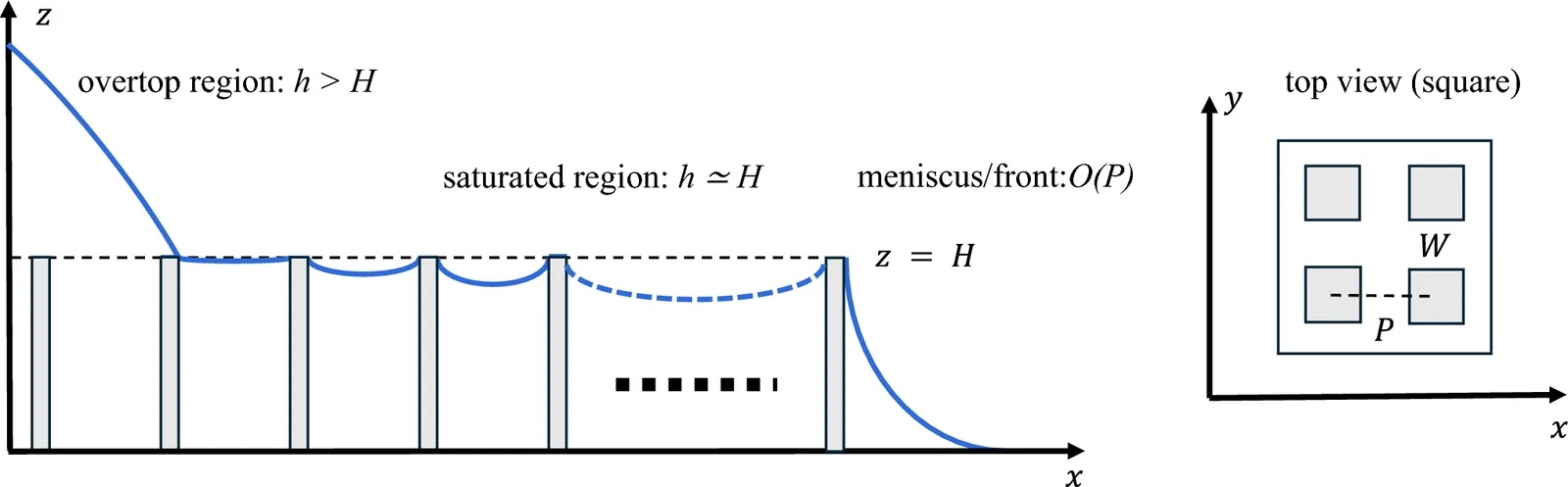
Liquid film imbibing a periodic micropillar array: Behind the advancing front, the texture is saturated (h≃H), while further back the film may overtop the pillars (h>H); the front/meniscus region extent is of order of the pillar pitch. The pillar width is *W*, their height *H*, and the array is a square lattice of pitch *P*

In this work, we derive explicit analytical mobility expressions for both pressure-driven and Marangoni-driven mean flows through and over periodic micropillar arrays. We solve the Stokes equations in a representative unit cell mapped onto an equivalent open microchannel geometry, providing a direct microscopic derivation of the hydraulic conductance and clarifying how lateral confinement modifies the flow inside the texture. The resulting Fourier series solution yields explicit velocity fields and mobilities, while the truncation error of the series can be quantified systematically. We then match the flow in the textured region to a free liquid layer above the pillars, obtaining mobility laws valid both for confined films and for overtopped textures. In the weak confinement limit, the mobilities converge toward the free-flow thin-film behavior, whereas stronger confinement reveals three hydrodynamic regimes: a nearly free ultra-thin-layer regime, a confined regime controlled by the pillar spacing, and a thick-film regime in which the textured layer acts as an effective partial slip substrate.

The resulting mobility formulation provides the constitutive ingredient needed to extend lubrication theory to droplets and films spreading on pillar arrays under combined capillary and Marangoni driving. It recovers known hemiwicking limits and provides a framework for interpreting more complex wetting dynamics on textured substrates, including vapor-mediated wetting and imbibition control [28].

## Physical system and governing equations

**Fig. 2:**
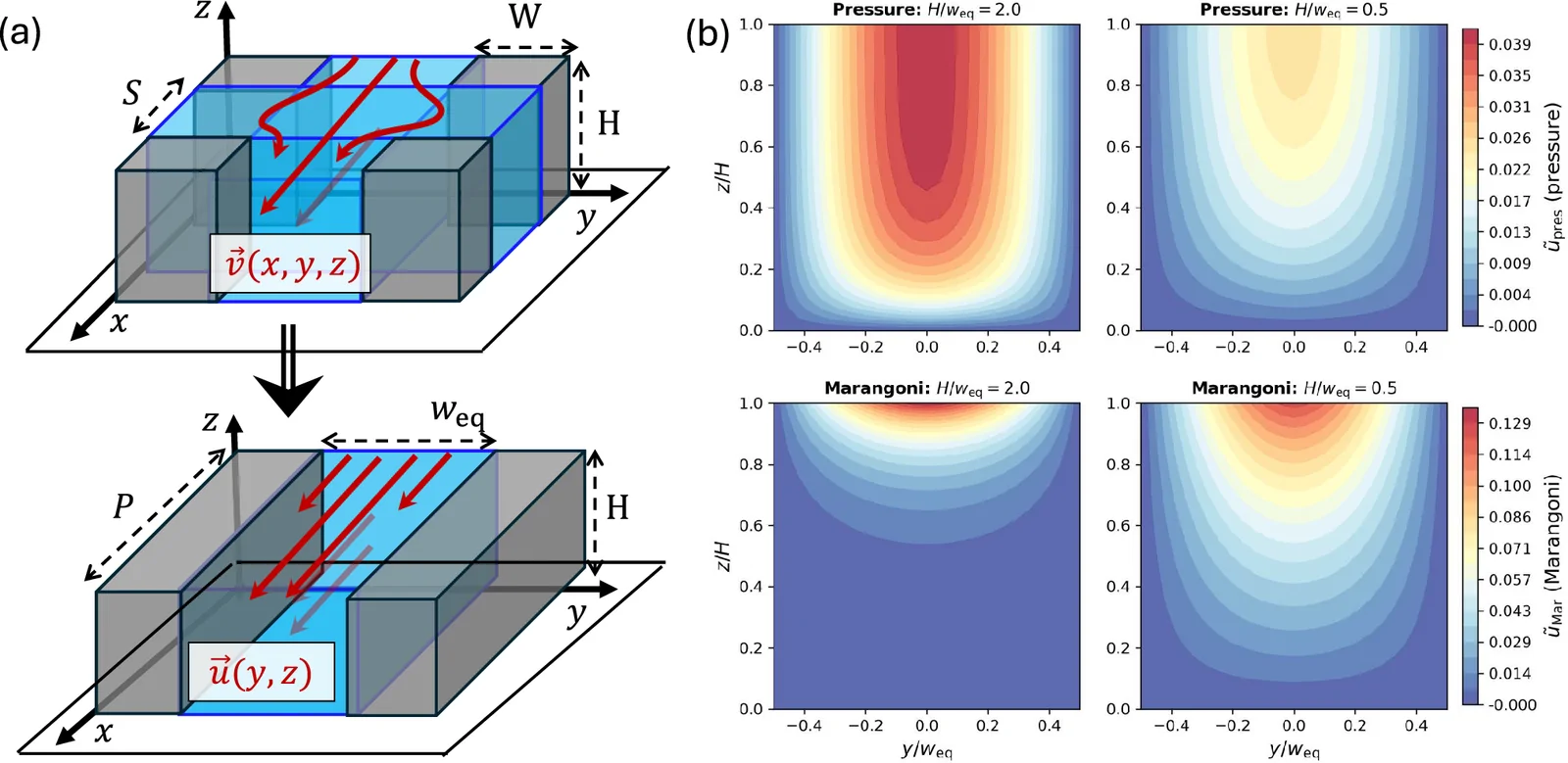
**a** Equivalent open microchannel used to approximate the flow through one unit cell of the pillar array (v→u, independent of the streamwise coordinate *x*) and compute the associated viscous dissipation. The texture is mapped onto a rectangular channel of height *H*, width $w_{\textrm{eq}}=\varepsilon P$, preserving the available liquid volume per unit cell. **b** Cross-sectional velocity fields $\tilde{u}(y,z)$ in the equivalent microchannel, for pressure-driven flow $\tilde{u}_{\textrm{pres}}=-\eta u_{\textrm{pres}}/(p_x w_{\textrm{eq}}^2)$ (top), and Marangoni-driven flow $\tilde{u}_{\textrm{Mar}}=\eta u_{\textrm{Mar}}/(\gamma _x w_{\textrm{eq}})$ (bottom), shown for different values of $H/w_{\textrm{eq}}$ with $w_{\textrm{eq}}=\varepsilon P$, using the reference Fourier series (n=200)

### Geometry and assumptions

We consider a square lattice of square micropillars on a flat substrate of pitch *P* (Fig. 1). Each pillar has width *W*, height *H*, and is separated from its neighbors by a gap

3 $$\begin{aligned} S = P - W. \end{aligned}$$

The corresponding projected solid fraction and porosity are

4 $$\begin{aligned} \phi _s = \frac{W^2}{P^2}, \qquad \varepsilon = 1-\phi _s. \end{aligned}$$

The roughness factor, defined as the ratio of the actual surface area to the projected surface area of a unit cell, is given by:

5 $$\begin{aligned} r = \frac{A_\textrm{total}}{A_\textrm{projected}} = 1 + \frac{4HW}{P^2}. \end{aligned}$$

where a perfectly flat surface yields r=1, whereas any real or engineered texture gives r>1. This purely geometric factor scales the interfacial free energy and thereby sets both the static and dynamic wetting properties of textured surfaces: It relates the apparent contact angle to that on the flat surface (Wenzel model), and sets the capillary force that drives liquid into the texture [7].

We consider a liquid film of thickness *h*(*x*, *y*, *t*), occupying the textured region and, when relevant, extending above the pillar tops. The present derivation concerns the effective mobilities of this idealized film configuration, assuming a flat mean interface within the texture. We do not resolve pillar-scale menisci or contact angle effects explicitly, which would presumably cause subdominant corrections to the mobilities derived here. An imposed in-plane pressure gradient is considered, independent of *z* at leading order within the lubrication approximation, as well as an imposed tangential stress at the upper liquid–gas interface to account for Marangoni forcing. The flow is assumed to be in the creeping flow regime, corresponding to vanishing Reynolds number, so that inertia is negligible and the response is linear in the applied forcing. No slip is imposed at all solid boundaries, i.e., on the substrate and the pillar sidewalls.

Two configurations are considered. In the first, the liquid remains confined within the texture, so that h≤H. In the second, the film overtops the pillars and a free liquid layer develops above the textured region, so that h>H. The derivation below is constructed to connect these two regimes continuously.

### Equivalent microchannel construction

Inspired by previous equivalent channel-based descriptions of viscous dissipation in textured wicking [25–27], we approximate the flow field $\overrightarrow{v}$ in a single unit cell by the field $\overrightarrow{u}$ in an equivalent open rectangular microchannel. As illustrated in Fig. 2a, the mapping is onto a microchannel of the same height *H* and width

6 $$\begin{aligned} w_{\textrm{eq}}=\varepsilon P, \end{aligned}$$

obtained by matching the liquid volume per unit streamwise length. The resulting approximated flow field $\overrightarrow{u}$ then becomes independent of the streamwise coordinate ($\overrightarrow{v}(x,y,z)\rightarrow \overrightarrow{u}(y,z)$, see Fig. 2a). Within this representation, the geometric lateral scales *S* and *W* are replaced by the equivalent width $w_{\textrm{eq}}$.

This construction preserves the liquid volume available in the textured unit cell while providing a direct route to the associated viscous resistance. It also yields an explicit geometry-based mobility without introducing permeability or other effective medium parameters as independent inputs.

### Governing equations in the equivalent channel

Within the equivalent microchannel, we assume a fully developed unidirectional flow

7 $$\begin{aligned} \textbf{u}=u(y,z)\,\textbf{e}_x, \end{aligned}$$

driven by a pressure gradient

8 $$\begin{aligned} \frac{\partial p}{\partial x}=p_x \end{aligned}$$

and, at the upper interface, by a surface tension gradient

9 $$\begin{aligned} \frac{\partial \gamma }{\partial x}=\gamma _x, \end{aligned}$$

both taken to be constant at the scale of the unit cell, so that the velocity has no *x*-dependence. The Stokes equations then reduce to

10 $$\begin{aligned} \eta \left( \frac{\partial ^2 u}{\partial y^2} +\frac{\partial ^2 u}{\partial z^2}\right) =p_x, \quad \frac{\partial p}{\partial y}= \frac{\partial p}{\partial z}=0. \end{aligned}$$

The boundary conditions are no slip on the sidewalls and at the bottom wall, together with a tangential stress condition at the upper interface:

11 $$\begin{aligned} u\!\left( \pm \frac{w_{\textrm{eq}}}{2},z\right) = u(y,0)=0, ~ \eta \frac{\partial u}{\partial z}(y,h)=\gamma _x. \end{aligned}$$

For purely pressure-driven flow, $\gamma _x=0$, so that the upper boundary is shear-free. For purely Marangoni-driven flow, $p_x=0$. More generally, both forcings may act simultaneously.

**Fig. 3:**
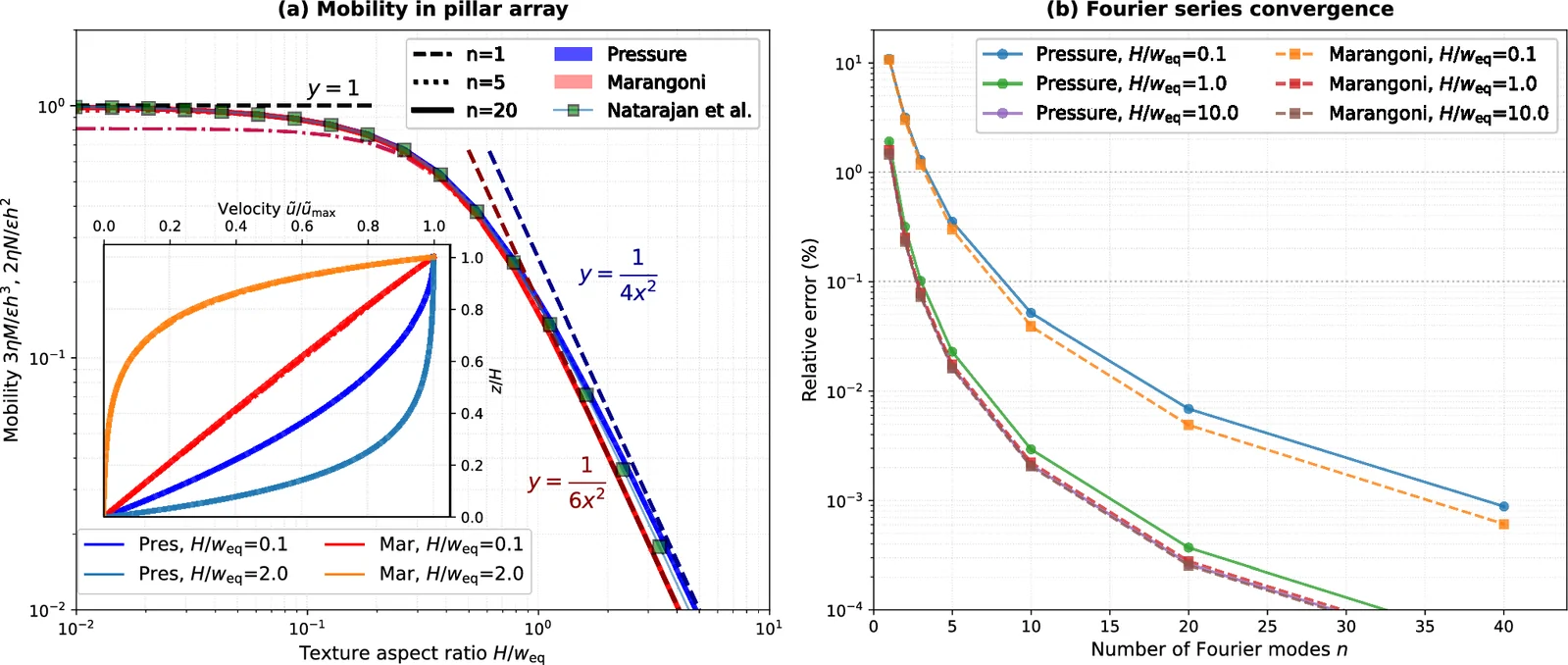
Mobility in pillar arrays and Fourier series convergence. **a** Dimensionless mobility $\tilde{M}$ (pressure-driven, blue-toned lines) and $\tilde{N}$ (Marangoni-driven, red-toned lines) as functions of the texture aspect ratio $H/w_{\textrm{eq}}$ in the confined pillar region, shown for three truncation levels. The asymptotic mobilities are shown as dashed lines (expressions (21) in dark red and blue for high $H/w_{\textrm{eq}}$, expressions (22) in black for small $H/w_{\textrm{eq}}$). The single-mode equivalent channel result of Natarajan et al. [25] is also shown for comparison. Inset: vertical velocity profiles $\tilde{u}(z)$ at h=H for $H/w_{\textrm{eq}}=0.1$ and $H/w_{\textrm{eq}}=10$, for both pressure-driven and Marangoni-driven flows with truncations n=1,5,20 (dash-dotted, dotted, solid, nearly overlapping). **b** Relative error in the computed mobility as a function of the number of retained Fourier modes for pressure-driven and Marangoni-driven flows for varying $H/w_{\textrm{eq}}$. The convergence is rapid over the full parameter range, with errors below 1% at n=5 and below 0.1% at n=10. Parameters: ε=0.5

### Flow fields and integrated mobilities

Equation (10) is a Poisson equation for $p_x\ne 0$ and reduces to Laplace’s equation when $p_x=0$. It is solved by separation of variables, expanding the solution in a Fourier series that automatically satisfies the sidewall boundary conditions (11). The resulting velocity fields in the channel cross section are:

12 $$\begin{aligned}  &   u_{\textrm{pres}}(y,z)= -\frac{p_x}{\eta } \sum _{n=1,3,5,\ldots } (-1)^{\frac{n-1}{2}} \frac{4\ell _n^2}{n\pi } \nonumber \\  &   \quad \left[ 1-\frac{\cosh \!\left( \frac{z-h}{\ell _n}\right) }{\cosh \!\left( \frac{h}{\ell _n}\right) } \right] \cos \!\left( \frac{y}{\ell _n}\right) , \end{aligned}$$

for pressure-driven flow, and

13 $$\begin{aligned}  &   u_{\textrm{Mar}}(y,z)= \frac{\gamma _x}{\eta } \sum _{n=1,3,5,\ldots } (-1)^{\frac{n-1}{2}} \frac{4\ell _n}{n\pi } \nonumber \\  &   \quad \frac{\sinh \!\left( \frac{z}{\ell _n}\right) }{\cosh \!\left( \frac{h}{\ell _n}\right) } \cos \!\left( \frac{y}{\ell _n}\right) , \end{aligned}$$

for Marangoni-driven flow. Only odd modes appear by symmetry. Each mode is associated with a lateral confinement length

14 $$\begin{aligned} \ell _n=\frac{w_{\textrm{eq}}}{n\pi } =\frac{\varepsilon P}{n\pi }, \end{aligned}$$

which couples the velocity field to the lateral size of the unit cell. In the effective medium interpretation developed later, the first mode scale $\ell _1$ plays the role of a Brinkman-like screening length [33, 34] (see Supplementary section S3). For generality, the local fluid thickness in the equivalent channel is written as *h*, so that the same modal expressions apply both to the confined case h≤H and, later, to the lower-layer contribution in the matched two-layer formulation. The detailed derivation is given in Supplementary section S1.

The corresponding pressure-driven and Marangoni-driven flow fields are shown in Fig. 2b. In the strong confinement limit $H/{w_{\textrm{eq}}} \gg 1$, the flow becomes strongly shaped by lateral confinement imposed by the texture. With increasing confinement, pressure-driven flow develops a Poiseuille profile in the lateral direction, while developing a (half-)plug profile in the *z*-direction, except for a boundary layer close to the substrate. Marangoni-driven flow remains confined at the free interface because the shear cannot penetrate significantly beyond a depth of order $w_{\textrm{eq}}$. The equivalent microchannel approach thus captures how the same lateral confinement affects bulk-driven and surface-driven transport in qualitatively different ways.

The volumetric flow rate through the equivalent channel is

15 $$\begin{aligned} Q=\int _{-w_{\textrm{eq}}/2}^{w_{\textrm{eq}}/2}\int _0^h \left[ u_{\textrm{pres}}(y,z) + u_{\textrm{Mar}}(y,z)\right] \,dz\,dy. \end{aligned}$$

Using the thin-film convention, the averaged flux per unit transverse (*y*) length is then defined as

16 $$\begin{aligned} q=\frac{Q}{P}, \end{aligned}$$

which introduces the pressure-driven and Marangoni-driven mobilities through

17 $$\begin{aligned} q=-M(h,P,\varepsilon )\,p_x+N(h,P,\varepsilon )\,\gamma _x. \end{aligned}$$

Integrating Eqs. (12) and (13) over the channel cross section yields

18 $$\begin{aligned} M(h,P,\varepsilon )= \sum _{\begin{array}{c} n=1\\ n\ \textrm{odd} \end{array}}^{\infty } \frac{8\varepsilon \ell _n^2}{\eta \pi ^2 n^2} \left[ h-\ell _n\tanh \!\left( \frac{h}{\ell _n}\right) \right] , \end{aligned}$$

and

19 $$\begin{aligned} N(h,P,\varepsilon )= \sum _{\begin{array}{c} n=1\\ n\ \textrm{odd} \end{array}}^{\infty } \frac{8\varepsilon \ell _n^2}{\eta \pi ^2 n^2} \left[ 1-{{\,\textrm{sech}\,}}\!\left( \frac{h}{\ell _n}\right) \right] . \end{aligned}$$

These expressions already reveal the key difference between the two forcings. In Eq. (18), the pressure-driven mobility contains an additional factor of the liquid thickness h, reflecting that the pressure gradient drives flow through the bulk volume. By contrast, Eq. (19) shows that the Marangoni mobility is controlled by surface forcing transmitted downward by viscosity and therefore does not acquire the same explicit term in h. The corresponding asymptotic limits are shown below, and their detailed derivation is given in Supplementary Information sections S1.2 and S1.1.

In the laterally confined limit,

20 $$\begin{aligned} h \gg \ell _1 \sim \varepsilon P \implies \forall n, h \gg \ell _n. \end{aligned}$$

For each mode, the bracketed term in equations (18) and (19) approaches *h* and 1, respectively, leaving a sum over odd *n* that decays as $n^{-4}$ and converges to $\pi ^4 / 96$. The mobilities then recover the standard solution for flows confined in a rectangular channel [35] multiplied by the porosity,

21 $$\begin{aligned} M \sim \frac{\varepsilon h(\varepsilon P)^2}{12\eta }, \qquad N \sim \frac{\varepsilon (\varepsilon P)^2}{12\eta }. \end{aligned}$$

Thus, the pressure-driven mobility continues to grow linearly with h, whereas the Marangoni mobility saturates once the liquid thickness exceeds the confinement scale.

In the weak confinement limit $h \ll \ell _1$, the film is much thinner than the lateral cell and therefore barely senses the sidewalls, so that the texture acts only through the porosity factor and the flow reduces to a porosity-reduced free film. In modal terms, each mode does not lie in its own wide-channel regime since high enough modes satisfy $h > rsim \ell _n = \ell _1/n$. Nonetheless, since the modal weights decay as $n^{-4}$, the convergence of the series is exact at leading order (see SI sections S1.2 and S1.1): The fundamental mode already carries the full result, while the finer lateral modes (scale $\ell _1/n$) are unresolved by so thin a film and rapidly suppressed. Since for all modes with $h \ll \ell _n$ the bracketed term in equations (18) and (19) reduces to $h^{3}/(3 \ell ^2_n)$ and $h^{2}/(2 \ell ^2_n)$, respectively, one obtains the asymptotic mobilities:

22 $$\begin{aligned} M \approx \frac{\varepsilon h^3}{3\eta }, \qquad N \approx \frac{\varepsilon h^2}{2\eta }, \end{aligned}$$

which corresponds to the standard thin-film forms multiplied by the porosity.

Figure 3a shows the complete mobility solutions (18) and (19) at different truncations. The porosity-reduced thin-film limit and laterally confined limit are both recovered asymptotically (dashed lines in Fig. 3a, $3 \eta M / \varepsilon h^3,~ 2 \eta N / \varepsilon h^2 \rightarrow 1$ for $H\ll \ell _1$ and $\sim 1\,/4 x^2,~\sim 1\,/6 x^2$, respectively, with $x= h/w_{\textrm{eq}}$ for $H\gg \ell _1$). They show a crossover at the texture aspect ratio $H/\ell _1$. Correspondingly, the flow profiles transition from Poiseuille-like (pressure-driven) and Couette-like (Marangoni) in the weak confinement limit to (half-)plug-like and surface-localized in the laterally confined limit. This transition is recovered already at low truncation order, showing that only a few odd Fourier modes are needed to qualitatively reproduce the full solution.

Figure 3b shows the convergence of the Fourier series approximation. The modal weights scale as $\ell _n^2/n^2 (\sim (\varepsilon P)^2/n^4)$, so the first mode captures the dominant contribution, and retaining only the first three odd modes (n=1,3,5) already yields errors below 1% across the full parameter range. Particularly at moderate and large confinement, this justifies that using only the first terms of the exact series for the mobility already provides an excellent approximation.

The present formulation recovers the leading-order equivalent channel result of Natarajan et al. [25]. A quantitative comparison against reported hemiwicking data is also provided in the Supplementary Information section S4.1.

**Fig. 4:**
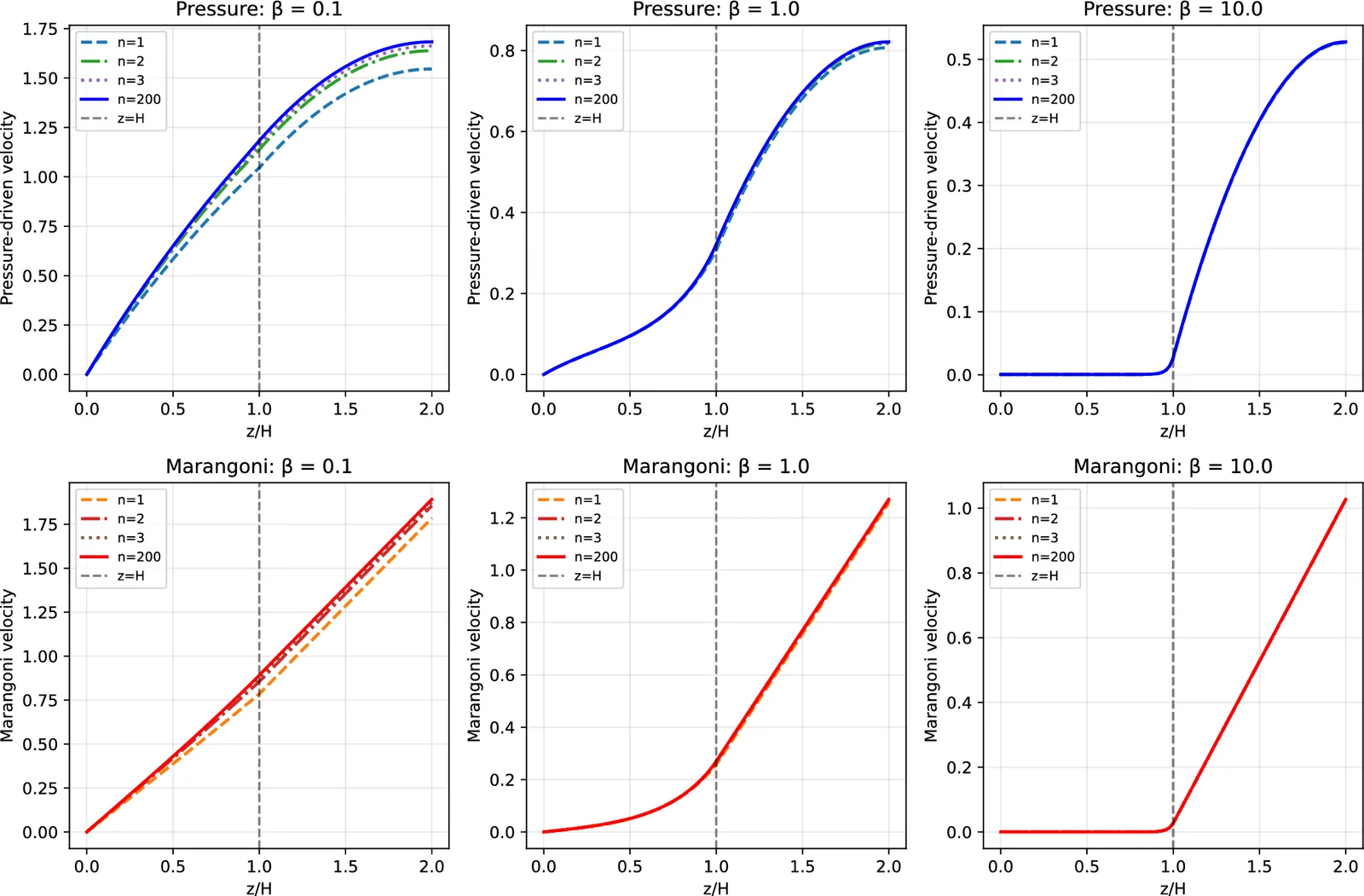
Rescaled velocity profiles for pressure-driven $\tilde{u}_{\textrm{pres}}=-\eta u_{\textrm{pres}}/(p_x H^2)$ and Marangoni-driven flows $\tilde{u}_{\textrm{Mar}}=\eta u_{\textrm{Mar}}/(\gamma _x H)$ in the two-layer regime (the lower layer uses $\bar{u}_1$, the upper layer $u_2$) for varying values of $H/w_{\textrm{eq}}$ with ε=0.5. The profiles illustrate the strong dissipation within the textured region and the redevelopment of the flow above the pillar tops (matched two-layer solutions; see Supplementary section S1.4)

## General mobility expressions for arbitrary film thickness

When the liquid film overtops the pillars, i.e., for h>H, the flow can be naturally decomposed into two regions: a lower confined layer within the texture and an upper, free liquid layer above the pillar tops. The lower region retains the confinement imposed by the micropillar array, whereas the upper region behaves as a classical lubrication layer, though with a modified boundary condition, reminiscent of slip, at its bottom. The two-layer formulation is constructed by solving the flow in each region separately and matching velocity and tangential stress at the interface z=H.

Inside the textured region, 0<z<H, we retain the laterally averaged confined flow inherited from the microchannel formulation. The lower-layer velocity can therefore be written as

23 $$\begin{aligned} \bar{u}_1(z)  &   = \sum _{\begin{array}{c} n=1\\ n\ \textrm{odd} \end{array}}^{\infty } \frac{8\varepsilon \ell _n}{n^2\pi ^2\eta } \Bigg [ -p_x\ell _n\left( 1-\cosh \!\left( \frac{z}{\ell _n}\right) \right) \nonumber \\  &   \quad + B_n \sinh \!\left( \frac{z}{\ell _n}\right) \Bigg ], \end{aligned}$$

where $B_n$ are matching coefficients to be determined. Here

24 $$\begin{aligned} \bar{u}_1(z)=\frac{1}{P}\int _{-w_{\textrm{eq}}/2}^{w_{\textrm{eq}}/2}u(y,z)\,dy, \quad w_{\textrm{eq}}=\varepsilon P, \end{aligned}$$

denotes the lateral average over the unit cell.

Above the pillars, in the region H<z<h, the flow obeys the classical Stokes–lubrication equation

25 $$\begin{aligned} \eta \frac{d^2u_2}{dz^2}=p_x, \qquad u_2'(h)=\frac{\gamma _x}{\eta }. \end{aligned}$$

The solution is

26 $$\begin{aligned} u_2(z)=\frac{p_x}{2\eta }z^2+\frac{\gamma _x-p_x h}{\eta }z+D, \end{aligned}$$

with the constant *D* fixed by velocity continuity at z=H.

The coupling between the two regions is obtained by imposing continuity of velocity,

27 $$\begin{aligned} \bar{u}_1(H)=u_2(H), \end{aligned}$$

together with tangential stress continuity at the top of the pillar layer. To write this matching in a form consistent with the laterally averaged lower-layer transport, we introduce an effective coefficient $\eta _{\textrm{eff}}$ through

28 $$\begin{aligned} \eta _{\textrm{eff}}\,\bar{u}_1'(H)=\eta \,u_2'(H). \end{aligned}$$

This coefficient accounts for the fact that the lower flow is represented here through a porosity-reduced, laterally averaged description rather than through the full local three-dimensional stress distribution. It is therefore not introduced as an independent constitutive parameter of the textured medium, but as the quantity required to make the matched two-layer formulation consistent with the mobilities in the pillar region. The detailed algebra and its identification are given in Supplementary sections S1.3 and S1.4.

The matched profiles are shown in Fig. 4. They highlight the central physical picture of the two-layer model: Within the texture, the flow remains strongly hindered by the microstructure, whereas above the pillar tops the velocity redevelops into the familiar parabolic or Couette-like profiles of pressure-driven and Marangoni-driven free-layer transport. As illustrated in Fig. 4, the balance between these two contributions is governed by the confinement ratio $H/w_{\textrm{eq}}$: Weak confinement leads to nearly free-film profiles across the full thickness, whereas stronger confinement localizes dissipation within the textured region and delays the redevelopment of the flow above the pillars.

This matched construction treats the upper free layer as laterally homogeneous, whereas immediately above the pillar tops the flow still carries the spanwise modulation of the confined layer. We compare in Supplementary Sec. S2 the derived flow profiles with full numerical solutions of the Stokes equations using finite-element methods [36]. Systematic overpredictions appear immediately above the pillar tops at strong confinement $H/w_{\textrm{eq}} > rsim 1$, because the matched construction does not account for the additional dissipation on the pillar tops, particularly for Marangoni-driven flows; in the weak confinement limit, the matched profiles instead always converge to the full solution.

This matched two-layer structure can then be integrated directly to obtain the effective mobility laws for films of arbitrary thickness.

Integrating the lower- and upper-layer velocity fields over their respective domains gives the total flux for h>H,

29 $$\begin{aligned} q= \int _{0}^{H}\bar{u}_1(z)\,dz + \int _{H}^{h}u_2(z)\,dz, \end{aligned}$$

which we identify with $q = -M(h)\,p_x + N(h)\,\gamma _x$.

For the arbitrary thickness result, we introduce the dimensionless quantities

30 $$\begin{aligned} \tilde{M}=\frac{3\eta }{H^3}M, \qquad \tilde{N}=\frac{2\eta }{H^2}N, \qquad \tilde{h}=\frac{h}{H}, \end{aligned}$$

together with the mode-dependent confinement parameter

31 $$\begin{aligned} \beta _n=\frac{H}{\ell _n} =\frac{n\pi H}{\varepsilon P}. \end{aligned}$$

For compactness, we define

$$\begin{aligned} m(\tilde{h})=\min (\tilde{h},1)\,, \qquad \Delta \tilde{h}=(\tilde{h}-1)\Theta (\tilde{h}-1)\,, \end{aligned}$$

with Θ(x) the Heaviside step function.

**Fig. 5:**
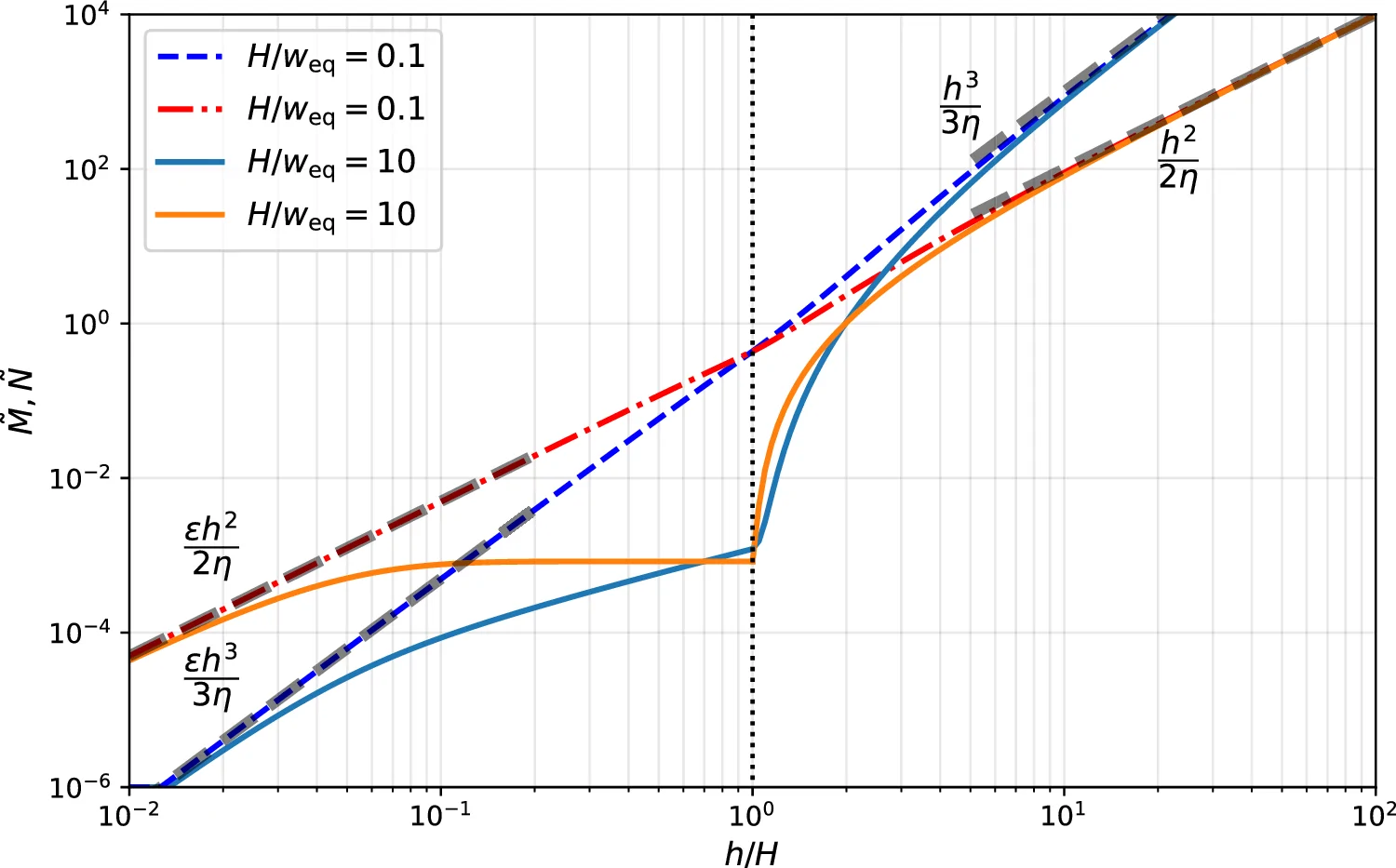
Rescaled pressure-driven mobility $\tilde{M}=(3\eta /H^3)M(h)$ and Marangoni-driven mobility $\tilde{N}=(2\eta /H^2)N(h)$ as functions of h/H for different values of $H/w_{\textrm{eq}}$, shown here for ε=0.5. The corresponding single-layer thin-film limits are also indicated in semitransparent dashed black lines and are recovered in the weak confinement regime ($H/w_{\textrm{eq}}\ll 1$)

The resulting mobilities valid for arbitrary film thickness are

32 $$\begin{aligned}  &   \tilde{M} = (\Delta \tilde{h})^3 + 3\sum _{\begin{array}{c} n=1,3,5,\ldots \end{array}} \frac{8\varepsilon }{n^2\pi ^2\beta _n} \nonumber \\  &   \quad \Bigg [ (\Delta \tilde{h})^2 \tanh \beta _n + 2\beta _n^{-1}\Delta \tilde{h}\,(1-{{\,\textrm{sech}\,}}\beta _n) \nonumber \\  &   \quad \hspace{7em} + \beta _n^{-2} \Big ( \beta _n\,m(\tilde{h}) - \tanh \!\big (\beta _n m(\tilde{h})\big ) \Big ) \Bigg ],\nonumber \\ \end{aligned}$$

33 $$\begin{aligned}  &   \tilde{N} = (\Delta \tilde{h})^2 + 2\sum _{\begin{array}{c} n=1,3,5,\ldots \end{array}} \frac{8\varepsilon }{n^2\pi ^2\beta _n} \nonumber \\  &   \quad \Bigg [ \Delta \tilde{h}\,\tanh \beta _n + \beta _n^{-1} \Big ( 1-{{\,\textrm{sech}\,}}\!\big (\beta _n m(\tilde{h})\big ) \Big ) \Bigg ], \end{aligned}$$

where $\Delta \tilde{h}=0$ and $m(\tilde{h})=\tilde{h}$ for $\tilde{h}<1$, whereas $\Delta \tilde{h}=\tilde{h}-1$ and $m(\tilde{h})=1$ for $\tilde{h}\ge 1$. These mobilities are shown in Fig. 5.

They are the main result of this paper, enabling an effective lubrication description of flow within and over pillar-decorated surfaces.

Their structure combines three contributions: a free-layer term $(\Delta \tilde{h})^3 \sim (h-H)^3/3$ (pressure) or $(\Delta \tilde{h})^2 \sim (h-H)^2/2$ (Marangoni), which dominates once a sufficiently thick liquid layer develops above the pillars; coupling terms, respectively $(\Delta \tilde{h})^2 \tanh \beta _n + 2\beta _n^{-1}\Delta \tilde{h}\,(1-{{\,\textrm{sech}\,}}\beta _n)$ (pressure) or $\Delta \tilde{h}\,\tanh \beta _n$ (Marangoni), which transfer momentum between the upper free layer and the hindered flow inside the texture; and a textured layer contribution $\sim \beta _n\,m(\tilde{h}) - \tanh \!\beta _n m(\tilde{h})$ (pressure) or $\sim 1-{{\,\textrm{sech}\,}}\!\beta _n m(\tilde{h})$ (Marangoni), which remains constant for h>H ($\sim H - \ell _n\tanh (H/\ell _n)$ (pressure) and $\sim 1-\operatorname {sech}(H/\ell _n)$ (Marangoni)) and preserves the signature of confined transport.

The continuity of the mobility functions at h=H follows by setting $\Delta \tilde{h} \rightarrow 0$ as $h\rightarrow H^+$ and $m(\tilde{h}) \rightarrow 1$ (i.e., $\tilde{h} \rightarrow 1$) as $h\rightarrow H^-$ in expressions (32) and (33). In this limit, the upper-layer and interlayer coupling terms all vanish, leaving only the textured layer contribution. Restoring dimensions recovers exactly the confined mobilities (18) and (19) for h<H. Remarkably, while the mobilities are continuous ($C^{0}$), they are not continuously differentiable at h=H. This slope change is physical: It marks the onset of an additional transport channel once the liquid rises above the pillar tops. In the weak confinement limit, the slope discontinuity vanishes as the texture ratio $H/w_{\textrm{eq}}\rightarrow 0$, whereas in the strong confinement limit it is exponentially suppressed for surface-driven flow (see Supplementary Information section S1.5).

**Fig. 6:**
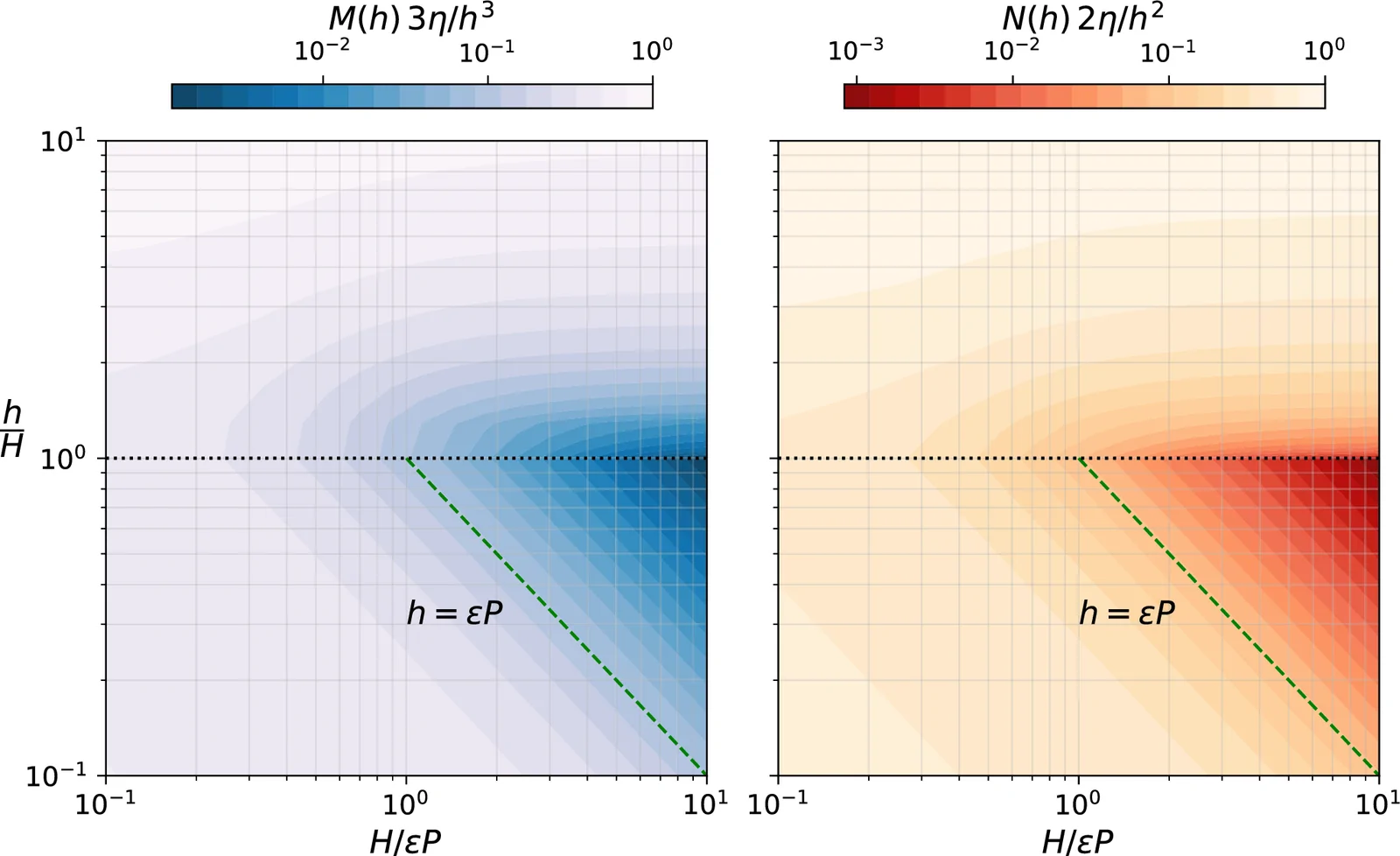
Regime map showing rescaled pressure-driven and Marangoni-driven mobilities for different values of *h*/*H* and H/εP (with ε=0.5), using the reference Fourier series (n=200). The green dashed line marks h=εP, where lateral confinement becomes dominant in the pillar layer and mobilities saturate

Figure 5 highlights the crossover from confined to free-layer transport. In the weak confinement limit, the mobilities rapidly collapse onto the single-layer thin-film behavior (porosity reduced in the texture layer) which is analytically retrieved in Supplementary Information section S1.6. On the other hand, for stronger confinement, the textured region leaves a pronounced dip relative to the non-textured mobilities over a broad range of film thicknesses.

Lastly, the hypothesis of laterally homogeneous flow for the top layer leads to an overestimation of the resulting flux, because it neglects a dissipation correction due to the pillar tops. This correction is confined to a layer of fixed thickness just above the pillar tops; it therefore vanishes for thick films and is bounded as $h\rightarrow H^+$, where the free layer itself shrinks to zero. We verify in Supplementary Sec. S2 that retaining the full lateral structure in the upper layer lowers the mobilities by only a few percent in the weak confinement limits, confirming the homogeneous approximation, while the overestimation of the model peaks at $\approx 20\%~(\varepsilon = 0.5)$ at intermediate and strong confinement. This correction is bounded and forcing-dependent: For pressure driving, it is suppressed at strong confinement, whereas for Marangoni driving it persists but confines increasingly at the pillar tops ($h/H \rightarrow 1^{+}$).

## Asymptotic interpretation and effective slip

The full expressions (32) and (33) make no assumption about the relative size of *H* and the confinement scale εP, and their ratio enters only through $\beta _n=n\pi H/(\varepsilon P)$, so that the mobilities remain valid for arbitrary H/εP. The comparison between *H* and εP does, however, govern the qualitative regime structure shown here in Fig. 6, which provides a global summary of the mobilities and their associated transport regimes.

The derived mobilities reveal three main regimes. For film thicknesses smaller than the lateral confinement scale, i.e., $h \lesssim O(\varepsilon P)$, the flow does not strongly feel the side walls of the texture and the mobilities remain close to those of an ordinary liquid layer, up to the porosity factor ε. In this weak confinement regime, the main effect of the pillar array is therefore geometrical rather than dissipative.

When the film thickness becomes of order εP while remaining below the pillar height, h<H, a second regime emerges in which lateral confinement inside the array controls the dissipation. More precisely, the dominant confinement scale is set by the first modal length

34 $$\begin{aligned} \ell _1=\frac{\varepsilon P}{\pi }, \end{aligned}$$

which governs the crossover from free-layer-like behavior to confined transport. In this regime, the pressure-driven mobility grows much more weakly with increasing thickness than in a free film, while the Marangoni-driven mobility approaches saturation. This stronger saturation reflects the fact that surface-driven shear penetrates only over a finite depth into the textured region, as already visible in the confined flow fields shown in Fig. 2b. Thus, textures may flip the dominance of pressure- and Marangoni-driven flows: Without textures, Marangoni flows typically outperform pressure-driven flows at very small film thicknesses, owing to their thickness scaling. This effect is exploited in Marangoni drying [37] and is of significant technological relevance. The surface-focusing effect of the textures, however, may render pressure-driven flows more efficient again, depending on the magnitude of the pressure and surface tension gradients and on $\ell _1$.

A third regime is reached when the liquid film overtops the pillars, h>H, and a free layer develops above the textured region. The hydrodynamics then result from the coupling of a lower, texture-confined layer and an upper lubrication layer. At sufficiently large film thickness, the transport progressively recovers the classical free-film behavior, with the textured region entering only through an effective slip length. Using

35 $$\begin{aligned} \Delta h=h-H, \end{aligned}$$

one finds, for $\Delta h \gg H$,

36 $$\begin{aligned} M&\sim \frac{\Delta h^3}{3\eta } + b_{\textrm{eff}}\,\frac{\Delta h^2}{\eta } + O(\Delta h), \end{aligned}$$

37 $$\begin{aligned} N&\sim \frac{\Delta h^2}{2\eta } + b_{\textrm{eff}}\,\frac{\Delta h}{\eta } + O(1). \end{aligned}$$

This identifies the effective slip length

38 $$\begin{aligned} b_{\textrm{eff}} = \sum _{n=1,3,5,\ldots } \frac{8\varepsilon \ell _n}{n^2\pi ^2} \tanh \!\left( \frac{H}{\ell _n}\right) , \end{aligned}$$

which is identical for both pressure-driven and Marangoni-driven transport. Thus, at large confinement ratios H/(εP), the pillar array acts as an effective slip boundary with

39 $$\begin{aligned} b_{\textrm{eff}}\sim \varepsilon \ell _1\tanh (H/\ell _1)\sim O(\varepsilon ^2 P). \end{aligned}$$

This asymptotic interpretation shows that the textured layer does not simply add a constant contribution to the mobility. Rather, it regularizes the boundary condition seen by the upper free film. In that sense, the micropillar array may be viewed as an effective slip substrate whose slip length is set by the dominant confinement scale of the textured region [32].

Hence, depending on the pillar array geometry, here quantified by the value of H/(εP), different transport regimes arise. The intermediate, saturated regime, where lateral confinement controls the dissipation, occupies the band $\ell _1\lesssim h\lesssim H$ and therefore emerges only when $\ell _1<H$. For $H\lesssim \varepsilon P$, the film overtops the pillars before lateral confinement becomes dominant. This corresponds to the weakly confining or relatively sparse textures where the mobilities rapidly approach those of a single free layer. For large values of H/(εP), the crossover is much more pronounced and the textured region leaves a strong signature over a broad range of film thicknesses. This is precisely the transition displayed in Fig. 6, where the saturated band opens only for H/εP>1, widening as the texture becomes more strongly confining.

## Discussion and conclusion

The mobility laws derived here provide a unified hydrodynamic description of liquid transport on micropillar arrays across confined hemiwicking, mixed confined/free-layer states, and fully overtopped films, for both pressure-driven and Marangoni-driven forcing. Their main contribution is therefore twofold: They provide the constitutive input required for thin-film modeling on textured substrates, and they clarify how the texture modifies the dominant mechanisms of viscous dissipation.

In the weak confinement limit, the ordinary thin-film regime is recovered above the pillar tops, while inside the textured region the same lubrication-type behavior is recovered up to the porosity factor ε. In that regime, the textured layer behaves as a porosity-reduced liquid film. Within a lubrication description, this is naturally accounted for by writing the local liquid volume variation as $\partial _t(\varepsilon h)$, so that the dynamics recover the standard thin-film form in terms of the accessible liquid volume. By contrast, in the strong confinement limit, a distinct transport regime emerges in which dissipation is controlled by the lateral confinement size of the texture. This leads to a much slower increase of the pressure-driven mobility with liquid thickness, and to a marked saturation of the Marangoni-driven mobility, since the induced flow remains strongly confined to a layer of depth εP at the free interface. In the overtopped regime, the same confined lower layer acts as an effective slip boundary for the free layer above the texture.

The present formulation therefore connects three hydrodynamic regimes within a single framework: porosity-reduced thin-film transport, confined hemiwicking inside the texture, and free-layer flow above a textured substrate with an effective slip length. This connection is one of the main conceptual outcomes of the work: The various regimes of wetting dynamics encountered on textured surfaces do not require separate constitutive laws, but can be qualitatively described by a common mobility formulation spanning the full range of film thicknesses.

Vapor-mediated filling of textures can thus cause switching or adaptation of the substrate and its wetting properties, from pinning or hemiwicking to a lubricated slippery surface. In this respect, the rigid micropillar array behaves as a controlled model of a switchable and adaptive substrate, with a purely geometric coupling between the liquid and the substrate state through different liquid transport mechanisms. The present hydrodynamic description therefore constitutes an additional step to understand dynamic wetting on adaptive, flexible, and switchable surfaces, where wetting and substrate dynamics are strongly coupled and jointly set the dissipation [1]. Future work will address this responsive behavior in wetting configurations, i.e., by combining the mobilities derived here with a lubrication-based solution of the wetting of volatile liquids on textured surfaces.

The same framework applies to both pressure-driven and Marangoni-driven flows. This is particularly relevant in situations where capillary transport competes with surface tension gradients, such as evaporation-induced composition gradients, thermal forcing, or vapor-mediated condensation. The present mobility formulation therefore provides a natural hydrodynamic basis for lubrication-type descriptions of more complex wetting states on textured surfaces, including droplet–film coexistence and vapor-mediated wetting control (see Supplementary Information section S4.2).

The two-layer matching also admits an effective medium interpretation. In that view, the dominant confinement scale $\ell _1=\varepsilon P/\pi$ plays the role of a momentum-screening length for transport within the textured region. In this limit, the formulation connects naturally to Darcy–Brinkman-type descriptions [33, 34] while retaining the higher-order corrections of the full Fourier series solution (see Supplementary Information section S3). Thus, the two-layer construction, composed of a dissipative, permeable lower layer matched to a free film is not specific to rigid pillars. Replacing the textured layer by another permeable medium amounts to modifying the confinement length by the relevant screening length, while the matching to the upper film and the effective slip picture are preserved. Lubricant-infused surfaces, where the sublayer transport acts through a mobile phase [6], or soft, permeable layers such as polymer brushes and gels therefore constitute natural candidates to extend this framework.

The derivation nonetheless relies on simplifying assumptions. First, the textured unit cell is represented by an equivalent open microchannel, which captures the dominant confinement physics but does not resolve the full three-dimensional recirculation around the exact pillar geometry. Second, the derivation assumes a flat interface inside the textured region and therefore does not resolve the detailed meniscus geometry within the unit cell, even though such menisci emerge spontaneously at vanishing speed for complete wetting. Third, it does not resolve the advancing imbibition front of an initially dry texture. In this region, where curvature and contact line dynamics both occur on the scale of the pillars, a more complex solution, probably beyond the limitations of long-wave approximation, would be more appropriate. Fourth, for an overtopping film, the matching procedure neglects the lateral inhomogeneity of the flow induced by viscous dissipation on the pillar tops. A comparison of the two-layer matched solutions with full numerical velocity fields in an equivalent geometry shows that the resulting overestimation peaks at $\approx 20\%~(\varepsilon = 0.5)$ for intermediate confinement and thin overtopping films. At strong confinement the two forcings differ: The pressure-driven deviation vanishes as the screened lower layer presents a uniform plane at z=H, while the Marangoni-driven deviation persists and concentrates at $h/H\rightarrow 1^+$. As this assessment is two-dimensional, it conservatively overestimates the heterogeneity of the true three-dimensional texture, so the homogeneous upper layer remains an accurate approximation over a wide range of parameters. For these reasons, the present mobility laws should be viewed as the hydrodynamic backbone of an effective/qualitative reduced-complexity description rather than as a full account of all microscopic details.

Despite these limitations, the present framework provides the constitutive ingredients needed to extend lubrication theory to droplets and films interacting with micropillar arrays under combined capillary and Marangoni driving, provided that the horizontal scale of interest is much larger than the periodicity of the texture. By connecting confined transport, overtopped films, and effective slip behavior within a single analytical mobility formulation, it provides a unified basis for effective long-wave descriptions of complex wetting dynamics on textured surfaces.

## Supplementary Information

Below is the link to the electronic supplementary material.

Supplementary file 1 (pdf 940 KB)

## Data Availability

The Fourier series solutions and all data used to generate the figures are provided in the Supplementary Material.
