# Supplementary material for: Effective mobilities for thin-film flows on micropillar arrays
Source: Eur Phys J E Soft Matter. 2026 Jul 31;49(8):68. doi: 10.1140/epje/s10189-026-00615-6 (PMC13427955; doi:10.1140/epje/s10189-026-00615-6)
Supplement: Supplementary file 1 — (pdf 940 KB) [file 10189_2026_615_MOESM1_ESM.pdf]

Effective Mobilities for Thin-Film Flows on Micropillar Arrays

Raphael Saiseau[1*] and Stefan Karpitschka[1]

[1*]Department of Physics, University of Konstanz, Konstanz, 78457, Germany.

*Corresponding author(s). E-mail(s): raphael.saiseau@uni-konstanz.de;

Keywords: Hemiwicking · Micropillar arrays · Thin-film dynamics · Marangoni flows · Lubrication theory · Textured surfaces

Supplementary Information

S1 Microchannel derivation

We consider an equivalent open microchannel of width

$$w_{\mathrm{eq}} = \varepsilon P \tag{S1}$$

and local fluid thickness h. The Stokes problem in the channel cross-section reads

$$\eta\left(\frac{\partial^2 u}{\partial y^2} + \frac{\partial^2 u}{\partial z^2}\right) = p_x, \tag{S2}$$

with the following boundary conditions

$$u\Big(\pm\frac{w_{\mathrm{eq}}}{2}, z\Big) = 0, \qquad u(y,0) = 0, \tag{S3}$$

and either

$$\partial_z u(y,h) = 0 \tag{S4}$$

for pressure-driven flow or

$$\eta\, \partial_z u(y,h) = \gamma_x \tag{S5}$$

for Marangoni-driven flow.

We expand the velocity field as

$$u(y,z) = \sum_{n=1}^{\infty} f_n(z) \cos\left(\frac{n\pi y}{w_{\mathrm{eq}}}\right). \tag{S6}$$

Only odd modes contribute, with Fourier coefficients

$$b_n = \begin{cases} (-1)^{(n-1)/2} \dfrac{4}{n\pi}, & n \text{ odd}, \\ 0, & n \text{ even}, \end{cases} \tag{S7}$$

and modal wavelengths

$$\ell_n = \frac{w_{\mathrm{eq}}}{n\pi}. \tag{S8}$$

For pressure-driven flow, each mode satisfies

$$f_n'' - \ell_n^{-2} f_n = \frac{p_x}{\eta} b_n. \tag{S9}$$

Imposing $f_n(0) = 0$ and $f_n'(h) = 0$ gives

$$f_n(z) = -\frac{p_x \ell_n^2}{\eta} b_n \left[1 - \frac{\cosh\left(\frac{z-h}{\ell_n}\right)}{\cosh\left(\frac{h}{\ell_n}\right)}\right]. \tag{S10}$$

For Marangoni-driven flow, the modal problem becomes

$$f_n'' - \ell_n^{-2} f_n = 0, \quad f_n(0) = 0, \quad f_n'(h) = \frac{\gamma_x}{\eta} c_n, \tag{S11}$$

where $c_n = b_n$ from the same cosine decomposition of the constant forcing. The corresponding solution is

$$f_n(z) = \frac{\gamma_x \ell_n c_n}{\eta} \frac{\sinh\left(\frac{z}{\ell_n}\right)}{\cosh\left(\frac{h}{\ell_n}\right)}. \tag{S12}$$

The complete velocity fields are therefore

$$u_{\mathrm{pres}}(y,z) = -\frac{p_x}{\eta} \sum_{n=1,3,5,\ldots} (-1)^{\frac{n-1}{2}} \frac{4 w_{\mathrm{eq}}^2}{n^3 \pi^3} \left[1 - \frac{\cosh\left(\frac{z-h}{\ell_n}\right)}{\cosh\left(\frac{h}{\ell_n}\right)}\right] \cos\left(\frac{n\pi y}{w_{\mathrm{eq}}}\right), \tag{S13}$$

for pressure-driven flow, and

$$u_{\mathrm{Mar}}(y,z) = \frac{\gamma_x}{\eta} \sum_{n=1,3,5,\ldots} (-1)^{\frac{n-1}{2}} \frac{4w_{\mathrm{eq}}}{n^2\pi^2} \frac{\sinh\left(\frac{z}{\ell_n}\right)}{\cosh\left(\frac{h}{\ell_n}\right)} \cos\left(\frac{n\pi y}{w_{\mathrm{eq}}}\right), \tag{S14}$$

for Marangoni-driven flow.

Integrating over the channel cross-section gives the pressure-driven volumetric flow rate

$$\begin{aligned} Q_p &= \int_{-w_{\mathrm{eq}}/2}^{w_{\mathrm{eq}}/2} \int_0^h u_{\mathrm{pres}}(y,z)\, dz\, dy \\ &= -p_x \sum_{\substack{n=1 \\ n \text{ odd}}}^{\infty} \frac{8w_{\mathrm{eq}}^3}{\eta\pi^4 n^4} \left[h - \frac{w_{\mathrm{eq}}}{n\pi} \tanh\left(\frac{n\pi h}{w_{\mathrm{eq}}}\right) \right], \end{aligned} \tag{S15}$$

and the Marangoni-driven flow rate

$$\begin{aligned} Q_M &= \int_{-w_{\mathrm{eq}}/2}^{w_{\mathrm{eq}}/2} \int_0^h u_{\mathrm{Mar}}(y,z)\, dz\, dy \\ &= \gamma_x \sum_{\substack{n=1 \\ n \text{ odd}}}^{\infty} \frac{8w_{\mathrm{eq}}^3}{\eta\pi^4 n^4} \left[1 - \operatorname{sech}\left(\frac{n\pi h}{w_{\mathrm{eq}}}\right) \right]. \end{aligned} \tag{S16}$$

Dividing by the unit-cell pitch P yields the microchannel mobilities used in the main text.

S1.1 Narrow-channel limit: $h \gg \varepsilon P$

For $h \gg \ell_n$, one has

$$\tanh\left(\frac{h}{\ell_n}\right) \to 1, \qquad \operatorname{sech}\left(\frac{h}{\ell_n}\right) \to 0. \tag{S17}$$

The mobilities then reduce to

$$M \approx \frac{8(\varepsilon P)^3 h}{\eta P \pi^4} \sum_{n=1,3,5,\ldots} \frac{1}{n^4} = \frac{\varepsilon^3 P^2 h}{12\eta}, \tag{S18}$$

and

$$N \approx \frac{8(\varepsilon P)^3}{\eta P \pi^4} \sum_{n=1,3,5,\ldots} \frac{1}{n^4} = \frac{\varepsilon(\varepsilon P)^2}{12\eta}. \tag{S19}$$

In this laterally confined limit, the transport reduces to that of a narrow microchannel of width w_{eq}, multiplied by the porosity.

S1.2 Wide-channel limit: $h \ll \varepsilon P$

For $h \ll \ell_n$, we use the expansions

$$\tanh\left(\frac{h}{\ell_n}\right) \approx \frac{h}{\ell_n} - \frac{1}{3}\left(\frac{h}{\ell_n}\right)^3, \tag{S20}$$

and

$$\operatorname{sech}\left(\frac{h}{\ell_n}\right) \approx 1 - \frac{1}{2}\left(\frac{h}{\ell_n}\right)^2. \tag{S21}$$

This gives

$$1 - \frac{\ell_n}{h}\tanh\left(\frac{h}{\ell_n}\right) \approx \frac{1}{3}\left(\frac{h}{\ell_n}\right)^2, \tag{S22}$$

and

$$1 - \operatorname{sech}\left(\frac{h}{\ell_n}\right) \approx \frac{1}{2}\left(\frac{h}{\ell_n}\right)^2. \tag{S23}$$

Substituting into the mobilities yields

$$M_{\text{press}} \approx \frac{8\varepsilon h^3}{3\eta\pi^2} \sum_{n=1,3,5,\ldots} \frac{1}{n^2} = \frac{\varepsilon h^3}{3\eta}, \tag{S24}$$

and

$$N_{\text{Mar}} \approx \frac{8\varepsilon h^2}{2\eta\pi^2} \sum_{n=1,3,5,\ldots} \frac{1}{n^2} = \frac{\varepsilon h^2}{2\eta}. \tag{S25}$$

Thus, in the weak-confinement limit, we recover the classical pressure-driven and Marangoni-driven thin-film mobilities multiplied by the porosity ε.

Strictly speaking, for a given h, sufficiently high order modes satisfy $\ell_n < h$. Writing $n_* \sim \ell_1/h$ for the crossover mode, the bracketed term in equations (18) and (19) reduces, for the lower modes $n \ll n_*$, to $h^3/(3\ell_n^2)$ and $h^2/(2\ell_n^2)$ respectively, while the higher modes $n \gg n_*$ give $h - \ell_n \simeq h$ and 1. Using the $n \ll n_*$ form for the lower modes and the $n \gg n_*$ form for the higher modes, expressions (18) and (19) are approximated by:

$$M_{\text{press}} \approx \frac{\varepsilon h^3}{3\eta} - \sum_{n>n_*,\text{odd}} \frac{8\varepsilon\ell_n^2}{\eta\pi^2 n^2}\left(\frac{h^3}{3\ell_n^2} - h\right),$$

and

$$N_{\text{Mar}} = \frac{\varepsilon h^2}{2\eta} - \sum_{n>n_*,\text{odd}} \frac{8\varepsilon\ell_n^2}{\eta\pi^2 n^2}\left(\frac{h^2}{2\ell_n^2} - 1\right).$$

At large n, the residual series behave as:

$$\sum_{n>n_*,\text{odd}} n^{-4}\left(\frac{n^2}{n_*^3} - \frac{1}{n_*}\right) \sim n_*^{-3} \sum_{n>n_*,\text{odd}} n^{-2},$$

$$\sum_{n>n_*,\text{odd}} n^{-4}\left(\frac{n^2}{n_*^2}-1\right) \sim n_*^{-2}\sum_{n>n_*,\text{odd}} n^{-2}.$$

As the odd-n series $\sum n^{-2}$ converges, the residuals are bounded for a given n_* and tend to zero as $n_* \to \infty$, confirming convergence of the full series to expressions (S24) and (S25) in the wide-channel limit.

S1.3 Matching conditions

For the overtopped case $h > H$, the lower microchannel-based solution is matched to a free layer above the pillars. At the interface $z = H$, we impose velocity continuity,

$$\bar{u}_1(H) = u_2(H), \tag{S26}$$

and tangential stress continuity in the form

$$\eta_{\text{eff}}\, \bar{u}_1'(H) = \eta\, u_2'(H), \tag{S27}$$

where η_{eff} is the effective matching coefficient introduced in the main text. The stress condition gives

$$\frac{\eta}{\eta_{\text{eff}}}\left[\gamma_x - p_x(h-H)\right] = \sum_{n\ \text{odd}} \frac{8\varepsilon\ell_n}{n^2\pi^2} \times \left[-p_x \sinh\left(\frac{H}{\ell_n}\right) + \frac{B_n}{\ell_n}\cosh\left(\frac{H}{\ell_n}\right)\right].$$

Using the closure relation

$$\sum_{n\ \text{odd}} \frac{8}{n^2\pi^2} = 1, \tag{S28}$$

we obtain

$$B_n = \frac{\eta}{\varepsilon\eta_{\text{eff}}}\left[\gamma_x - p_x(h-H)\right]\operatorname{sech}\left(\frac{H}{\ell_n}\right) - p_x\ell_n \tanh\left(\frac{H}{\ell_n}\right), \tag{S29}$$

which gives $\bar{u}_1$, the velocity field in the pillar region of the two-layer formulation.

To identify η_{eff}, we require that the two-layer formulation reduce continuously to the confined microchannel mobility in the limit $h \downarrow H$. This is most conveniently done for pure Marangoni forcing, for which the comparison isolates the interfacial shear transmission. Keeping only the Marangoni term, we use the two-layer flux $q(H)$ in the limit $h \downarrow H$:

$$q_M = \int_0^H \bar{u}_1 dz = \frac{\eta}{\varepsilon\eta_{\text{eff}}}\gamma_x \times \sum_{n\ \text{odd}} \frac{8\varepsilon\ell_n^2}{\eta\pi^2 n^2}\left[1 - \operatorname{sech}\left(\frac{H}{\ell_n}\right)\right], \tag{S30}$$

corresponding to the flux produced by a Marangoni stress exerted on the top layer immediately above the pillar tops. Comparing this with Q_M/P from expression (S16),

obtained from the Stokes solution in the confined pillar region, yields $\eta/(\varepsilon\eta_{\mathrm{eff}}) = 1$, i.e. $\eta_{\mathrm{eff}} = \eta/\varepsilon$.

S1.4 Complete velocity fields

Once the matching coefficient η_{eff} is identified from consistency with the confined flux, the complete two-layer solution can be written explicitly.

Pillar layer $(0 < z < H)$**.**
The laterally averaged lower-layer velocity is

$$\bar{u}_1(z) = \sum_{n=1,3,5,\ldots} \frac{8\varepsilon\ell_n}{n^2\pi^2\eta} \left\{ [\gamma_x - p_x(h-H)] \frac{\sinh\left(\frac{z}{\ell_n}\right)}{\cosh\left(\frac{H}{\ell_n}\right)} - p_x\ell_n \left[1 - \frac{\cosh\left(\frac{z-H}{\ell_n}\right)}{\cosh\left(\frac{H}{\ell_n}\right)} \right] \right\}. \tag{S31}$$

Free layer $(H < z < h)$**.**
The velocity in the upper layer is obtained from Eq. (26) with the integration constant fixed by continuity at $z = H$:

$$\begin{aligned} u_2(z) &= \frac{p_x}{2\eta}(z-H)^2 + \frac{\gamma_x - p_x(h-H)}{\eta}(z-H) \\ &+ \sum_{n=1,3,5,\ldots} \frac{8\varepsilon\ell_n}{n^2\pi^2\eta} \left\{ [\gamma_x - p_x(h-H)] \tanh\left(\frac{H}{\ell_n}\right) - p_x\ell_n \left[1 - \operatorname{sech}\left(\frac{H}{\ell_n}\right) \right] \right\}. \end{aligned} \tag{S32}$$

These are the velocity profiles plotted in Figure 4 for varying texture aspect ratios H/w_{eq}.

S1.5 Mobility functions continuity at the pillar tops

Setting $\Delta\tilde{h} \to 0$ as $\tilde{h} \to 1^+$ and $m(\tilde{h}) = \tilde{h} \to 1$ as $\tilde{h} \to 1^-$ in expressions (32) and (33) shows that the mobilities are continuous at $h = H$. Their derivatives, however, are governed by the modal confinement parameter $\beta_n = H/\ell_n = n\pi H/(\varepsilon P)$.

Differentiating the dimensional counterpart of expressions (32) and (33) above and below $h = H$, the first derivatives are

$$\begin{aligned} M'_- &= M'(H^-) = \sum_{n \text{ odd}} \frac{8\varepsilon\ell_n^2}{\eta\pi^2 n^2} \tanh^2\beta_n, \\ M'_+ &= M'(H^+) = \sum_{n \text{ odd}} \frac{8\varepsilon\ell_n^2}{\eta\pi^2 n^2} 2\big(1 - \operatorname{sech}\beta_n\big), \\ N'_- &= N'(H^-) = \sum_{n \text{ odd}} \frac{8\varepsilon\ell_n}{\eta\pi^2 n^2} \operatorname{sech}\beta_n \tanh\beta_n, \end{aligned}$$

$$N'_+ = N'(H^+) = \sum_{n \text{ odd}} \frac{8\varepsilon\ell_n}{\eta\pi^2 n^2} \tanh\beta_n.$$

The corresponding slope mismatches are:

$$M'_+ - M'_- = \sum_{n \text{ odd}} \frac{8\varepsilon\ell_n^2}{\eta\pi^2 n^2} \big(1 - \operatorname{sech}\beta_n\big)^2,$$
$$N'_+ - N'_- = \sum_{n \text{ odd}} \frac{8\varepsilon\ell_n}{\eta\pi^2 n^2} \tanh\beta_n \big(1 - \operatorname{sech}\beta_n\big),$$

both non-negative, so the free surface layer only increases the sensitivity of the mobilities to thickness.

Mode by mode, the slopes ratio is

$$\left.\frac{M'_+}{M'_-}\right|_n = \frac{2\,(1 - \operatorname{sech}\beta_n)}{\tanh^2\beta_n},$$
$$\left.\frac{N'_+}{N'_-}\right|_n = \cosh\beta_n,$$

leading to the relative jumps $\big[M'_+/M'_- - 1\big]_n = (1 - \operatorname{sech}\beta_n)^2/\tanh^2\beta_n$ and $\big[N'_+/N'_- - 1\big]_n = \cosh\beta_n - 1$.

Weak confinement.

Expanding for $\beta_n \to 0$, i.e. $H \ll \varepsilon P$,

$$\left.\frac{M'_+}{M'_-}\right|_n = 1 + \frac{\beta_n^2}{4} + \mathcal{O}(\beta_n^4),$$
$$\left.\frac{N'_+}{N'_-}\right|_n = 1 + \frac{\beta_n^2}{2} + \mathcal{O}(\beta_n^4).$$

The slopes therefore agree to leading order. Both mobilities are thus continuously differentiable to leading order. The pillar tops cease to be a distinguished height and the kink disappears.

Strong confinement.

In the opposite limit $\beta_n \to \infty$, i.e. $H \gg \varepsilon P$, $\tanh\beta_n \to 1$ and $\operatorname{sech}\beta_n \to 0$, so

$$\left.\frac{M'(H^+)}{M'(H^-)}\right|_n \to 2,$$
$$\left.\frac{N'(H^+)}{N'(H^-)}\right|_n = \cosh\beta_n \to \infty.$$

Here, the mobilities for the two driving mechanisms behave differently. For pressure driven flow, the slope simply *doubles* across the pillar tops. For Marangoni forcing the slope below the tops is exponentially suppressed, $N'(H^-) \sim 2\,e^{-\beta_n} \to 0$: the texture screens surface stresses. The mobility then rises sharply only once liquid overtops the pillars, reflecting a change of transport regime in strongly confining textures.

S1.6 Weak confinement limit of generalized mobility functions

We derive here an asymptotic expression of the generalized mobilities for $\beta_n \to 0$ ($H \ll \varepsilon P$). The convergence is justified in Supplementary section S1.2. Expanding the dimensional forms of expressions (32) and (33), every bracket becomes *independent of* n at leading order,

$$\begin{aligned} \ell_n \tanh \beta_n &\to H, \\ 2\ell_n^2 (1 - \operatorname{sech} \beta_n) &\to H^2, \\ \ell_n^2 \big(H - \ell_n \tanh \beta_n\big) &\to \frac{H^3}{3}, \end{aligned}$$

and the mode sum collapses using the closure relation. Regrouping gives the compact forms for the in-texture mobilities ($h \le H$)

$$M \to \frac{\varepsilon h^3}{3\eta}, \tag{S33}$$

$$N \to \frac{\varepsilon h^2}{2\eta}, \tag{S34}$$

as already obtained for the confined mobilities, expressions (S24) and (S25). For overtopping films ($h \ge H$), one obtains

$$M \to \frac{\varepsilon h^3}{3\eta} + (1-\varepsilon)\frac{(h-H)^3}{3\eta}, \tag{S35}$$

$$N \to \frac{\varepsilon h^2}{2\eta} + (1-\varepsilon)\frac{(h-H)^2}{2\eta}. \tag{S36}$$

These interpolate between a porosity-reduced free film inside the texture and the unobstructed free film above. At $\varepsilon = 1$ they collapse exactly to the flat-substrate films. The successive derivatives at $h = H$ are continuous up to

$$\begin{aligned} \big[M'''\big]_H &= \frac{2(1-\varepsilon)}{\eta}, \\ \big[N''\big]_H &= \frac{1-\varepsilon}{\eta}, \end{aligned}$$

so $M \in C^2$ and $N \in C^1$.

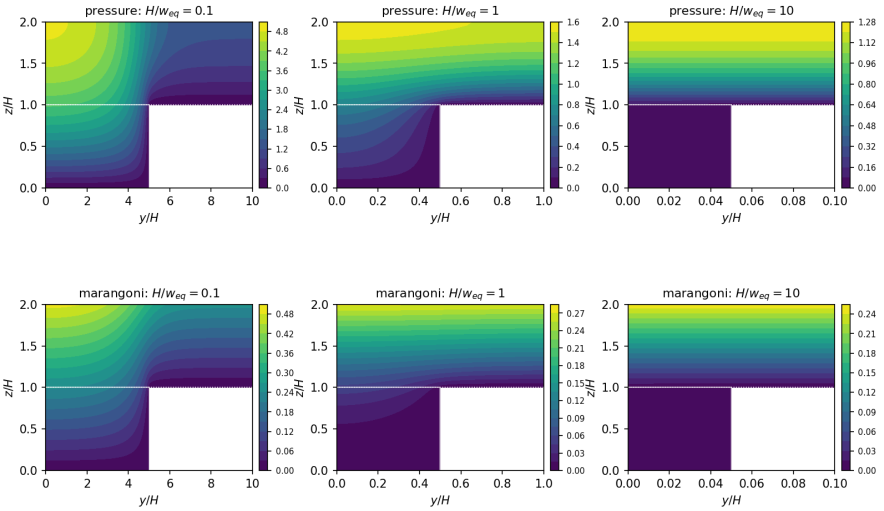

Fig. S2 Full computed two-dimensional velocity field on a half-microchannel equivalent unit cell (Figure S1), obtained using FEM (*pyoomph* software [1]). Velocities are reconstructed in SI units, using representative values $\eta = 2$ mPa s, $H = 10\,\mu$m, $p_x = 5 \times 10^4$ Pa m^{-1}, $\gamma_x = 5 \times 10^{-2}$ N m^{-2} ($\varepsilon = 0.5$).

S2 Finite-element validation of the matched two-layer model

Reduced problem and geometry

The matched two-layer mobilities (32) and (33) and flow velocities (S31) and (S32) treat the free layer above the pillars ($H < z < h$) as laterally homogeneous, whereas the flow there, at least close to the pillar tops, must be modulated by the texture geometry underneath. To quantify the error resulting from this assumption, we solve the full cross-sectional flow problem numerically, without lateral homogenization.

We solve the Stokes problem on the equivalent-microchannel half-cell, where it reduces to a two-dimensional problem. This equivalent-channel geometry maximizes the lateral inhomogeneity relative to the true three-dimensional pillar texture, in which the discrete pillars redistribute momentum laterally and further homogenize the flow above the tops. The comparison between the full numerical Stokes solutions and the matched two-layer model therefore overestimates the deviation due to the lateral-homogeneity assumption, and constitutes a conservative upper bound.

The Stokes equation is then solved on a stepped (L-shaped) half-cell, shown in Fig. S1, exploiting the mirror symmetry about the gap center ($y = 0$) and the pillar center ($y = P/2$), which makes it equivalent to the volume-preserving equivalent-channel mapping used in the main text. The resulting half-channel has width $w_{\mathrm{eq}}/2 = \varepsilon P/2$ for $0 < z < H$ and opens to the full half-pitch $P/2$ for $H < z < h$.

For fully developed, unidirectional flow $u(y,z)\,\hat{\mathbf{e}}_x$ the Stokes equations reduce to the scalar Poisson problem $\eta\,(\partial_{yy}+\partial_{zz})\,u = p_x$ with a stress-free condition at $z = h$ or a surface stress $\eta\,\partial_z u = \gamma_x$ for Marangoni flows (setting $p_x = 0$).

The problem is solved with quadratic (C^2) finite elements, using *pyoomph* [1], an object-oriented multi-physics framework based on *oomph-lib* [2] and *GiNaC* [3]. The grid is clustered toward the pillar corner at $(w_{\mathrm{eq}}/2, H)$. Figure S2 shows the numerical flow fields inside and above the texture for varying aspect ratios.

The lateral inhomogeneity of the flow immediately above the pillars is recovered numerically and is amplified at weak confinement, where the stress penetrates deeper into the texture. The evolution of this lateral variation with the texture ratio is shown in Fig. S3 with the corresponding laterally averaged profile $\bar{u}(z)$. Relative to the matched two-layer model, the difference vanishes in both limits, but for different reasons: for $H/w_{\mathrm{eq}} \gg 1$ the flow barely penetrates the texture and develops almost no lateral modulation, so the mean profile matches the model; for $H/w_{\mathrm{eq}} \ll 1$, the modulation persists but averages out, as the mismatch between the dissipation on the pillar tops and on the substrate weakens with decreasing confinement. The difference is therefore largest at intermediate confinement.

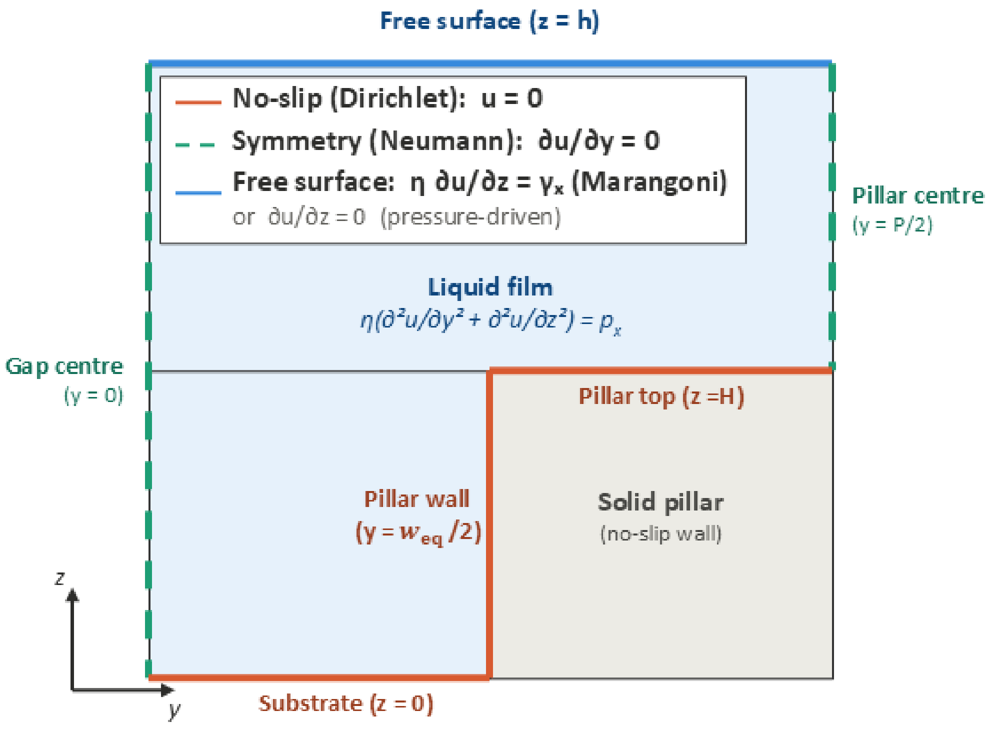

Fig. S1 Computational domain and boundary conditions for the numerical Stokes-flow problem. It is a half-microchannel-equivalent unit cell with mirror-symmetric lateral boundary conditions.

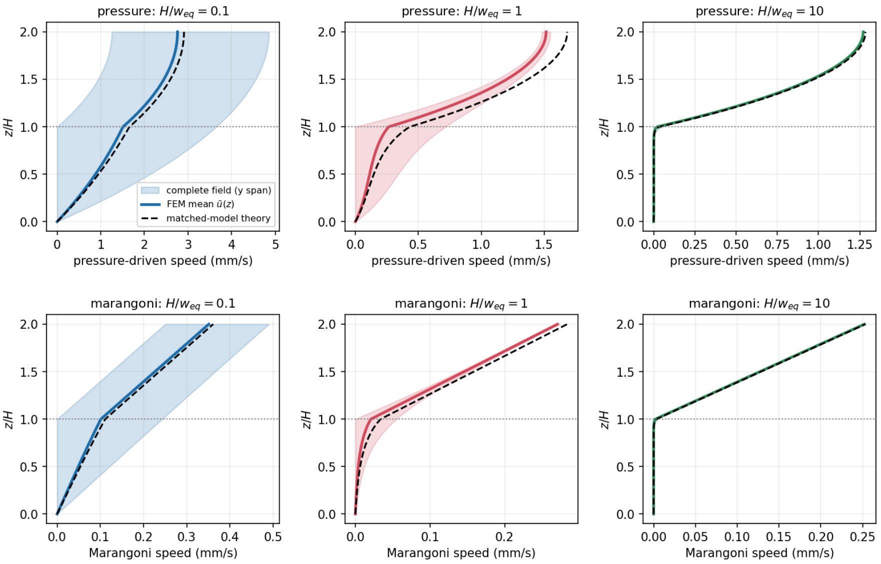

Fig. S3 Lateral-mean velocity profiles of the fields shown in Fig. S2, compared with the matched two-layer theory, expressions (S31) and (S32).

Numerical mobilities are then obtained from the integrated cross-sectional flux. Figure S4a compares the numerical and analytical mobilities as functions of h/H, spanning the sub-pillar ($h < H$) and overtopped ($h > H$) regimes, for both weak ($H/w_{\mathrm{eq}} = 0.1$) and strong ($H/w_{\mathrm{eq}} = 10$) confinement. The finite-element points lie on the analytical curves throughout, with the largest difference occurring, as expected, at moderate confinement $H/w_{\mathrm{eq}} \approx 1$ for films just above the pillar tops.

Figure S4b reports the relative deviation, above the pillar tops. It remains below $\simeq 22\%$ for $\tilde{M}$ and $\tilde{N}$, is non-monotonic, and corresponds to a few percent in both the weak-confinement limits. The two forcings separate at strong confinement: for pressure driving, screening renders the lower layer near-stagnant so that $z = H$ becomes a laterally uniform plane and the deviation collapses toward zero; for Marangoni driving, the interfacial stress keeps acting on the film layer confined at the pillar top, so the peak does not shrink but sharpens and migrates to $h/H \to 1^+$, mirroring the finite penetration depth of surface-driven shear. Strikingly, the lateral non-uniformity above the pillar tops decays as $\exp[-(z-H)/(P/2\pi)]$, i.e. over the fundamental lateral mode of the open cell, whose characteristic length is set by the pitch P rather than by the gap. The free layer therefore relaxes to a laterally uniform state within a finite, well-defined height above the texture, confirming the rapid screening of the heterogeneity anticipated above.

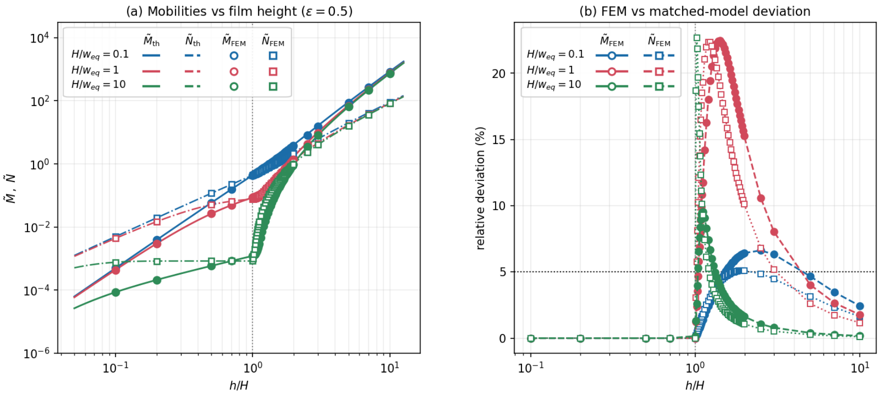

Fig. S4 (a) Rescaled mobilities for confinement ratios $H/w_{\mathrm{eq}} = 0.1, 1.0$ and 10 ($\varepsilon = 0.5$). Solid lines are expressions (32) and (33) of the main paper. Open symbols are direct numerical two-dimensional Stokes solutions obtained using FEM on the equivalent half-cell (see Fig. S1). The discrepancies between FEM and the theoretical curves are too small to be resolved on logarithmic scale. (b) Relative deviation between FEM and model.

S3 Darcy–Brinkman interpretation for effective medium description

S3.1 Films confined within the texture

As a secondary interpretation of the microchannel mobilities derived above, the textured region may be viewed as an effective Brinkman medium. For $h \le H$, we model the averaged velocity in the pillar layer by

$$\eta_{\mathrm{eff,B}}\, u'' - \frac{\eta}{\kappa} u = p_x, \qquad u(0) = 0, \tag{S37}$$

where $\eta_{\mathrm{eff,B}}$ is the effective viscosity associated with the Darcy–Brinkman description. Introducing the Brinkman screening length

$$\delta_B = \sqrt{\frac{\kappa \eta_{\mathrm{eff,B}}}{\eta}}, \tag{S38}$$

and imposing a shear-free upper boundary condition,

$$u'(h) = 0, \tag{S39}$$

we obtain

$$u(z) = -\frac{\kappa}{\eta} p_x \left[1 - \frac{\cosh\left(\frac{z-h}{\delta_B}\right)}{\cosh\left(\frac{h}{\delta_B}\right)} \right]. \tag{S40}$$

The corresponding flux is

$$q = -\frac{\kappa}{\eta}\left[h - \delta_B \tanh\left(\frac{h}{\delta_B}\right)\right] p_x, \tag{S41}$$

which yields the pressure-driven mobility

$$M_{\mathrm{in}}(h) = \frac{\delta_B^2 h}{\eta_{\mathrm{eff,B}}}\left[1 - \frac{\delta_B}{h}\tanh\left(\frac{h}{\delta_B}\right)\right]. \tag{S42}$$

For Marangoni forcing, imposing

$$\eta_{\mathrm{eff,B}}\, u'(h) = \gamma_x \tag{S43}$$

gives

$$N_{\mathrm{in}}(h) = \frac{\delta_B^2}{\eta_{\mathrm{eff,B}}}\left[1 - \mathrm{sech}\left(\frac{h}{\delta_B}\right)\right]. \tag{S44}$$

Matching the first Fourier mode of the microchannel problem yields the identifications

$$\kappa = \frac{8(\varepsilon P)^3}{P\pi^4}, \qquad \eta_{\mathrm{eff,B}} = \frac{\pi^2}{8\varepsilon}\eta, \qquad \delta_B = \frac{\varepsilon P}{\pi}. \tag{S45}$$

These expressions show that the relevant crossover scale inside the texture is not the pillar height itself, but a lateral screening scale controlled by the accessible void width.

The exact microchannel mobility is expressed as a sum over odd Fourier modes, each associated with a confinement length

$$\ell_n = \frac{\varepsilon P}{n\pi}. \tag{S46}$$

Because the modal weights decay rapidly with mode number, the first mode provides the dominant contribution over most of the parameter range. In that sense, the reduced effective-medium interpretation may be viewed as a first-mode approximation in which

$$\delta_B = \ell_1. \tag{S47}$$

This interpretation is useful for understanding the crossover behavior, but the full mobilities in the main text retain the higher-order corrections of the complete Fourier-series solution.

S3.2 Two-layer derivation for films overtopping the pillars

The same effective-medium viewpoint can be extended to the case $h > H$, where the flow is split into a lower Brinkman region $0 < z < H$ and an upper free Stokes region $H < z < h$. In the lower layer,

$$u_1(z) = \frac{\kappa}{\eta}p_x\left[\cosh\left(\frac{z}{\delta_B}\right) - 1\right] + B\sinh\left(\frac{z}{\delta_B}\right), \tag{S48}$$

whereas in the upper layer,

$$u_2(z) = \frac{p_x}{2\eta} z^2 + \frac{\gamma_x - p_x h}{\eta} z + D. \tag{S49}$$

Matching velocity ($u_1(H) = u_2(H)$) and tangential stress ($\eta_{\mathrm{eff,B}} u_1'(H) = \eta u_2'(H)$) at the pillar tops determines the coefficients, and integrating the two contributions gives the total flux

$$q = -M_{\mathrm{tot}}(h) p_x + N_{\mathrm{tot}}(h) \gamma_x. \tag{S50}$$

This yields

$$M_{\mathrm{tot}}(h) = \Theta(h-H) \left[\frac{(h-H)^3}{3\eta} + \frac{\delta_B \tanh \beta}{\eta_{\mathrm{eff,B}}} (h-H)^2 + 2\frac{\kappa}{\eta} (h-H)\,(1 - \operatorname{sech} \beta) \right] + \frac{\kappa}{\eta} \left[\min(h,H) - \delta_B \tanh\left(\frac{\min(h,H)}{\delta_B} \right) \right], \tag{S51}$$

and

$$N_{\mathrm{tot}}(h) = \Theta(h-H) \left[\frac{(h-H)^2}{2\eta} + \frac{\delta_B \tanh \beta}{\eta_{\mathrm{eff,B}}} (h-H) \right] + \frac{\kappa}{\eta} \left[1 - \operatorname{sech}\left(\frac{\min(h,H)}{\delta_B} \right) \right], \tag{S52}$$

with

$$\beta = \frac{H}{\delta_B}. \tag{S53}$$

These expressions are continuous at $h = H$ and reduce to the confined-layer mobilities, expressions (S42) and (S44) in the limit $h \downarrow H$. They correspond to the effective first-mode approximation of the full mobilities derived in the main text.

S4 Experimental validation and applications

S4.1 Quantitative comparison with hemiwicking experiments

In the confined-film region, where $h = H$, the mobility may be approximated by the saturated values $M_{\mathrm{sat}} = M(H)$ and, when relevant, $N_{\mathrm{sat}} = N(H)$. At the moving front, mass conservation gives

$$V_n = \frac{\mathbf{n} \cdot \mathbf{q}}{\varepsilon H}, \tag{S54}$$

so that the front speed is equal to the incoming flux divided by the locally stored liquid volume εH.

Within the energetic framework of rough wetting [4], the capillary pressure drop for partial wetting can be written, per unit invaded volume εH, as

$$\Delta p_{\mathrm{cap}} = \frac{\gamma}{\varepsilon H} \left[(r - \phi_s) \cos \theta_e - (1 - \phi_s) \right], \tag{S55}$$

with θ_e the Young contact angle, consistent with the usual hemiwicking criterion. In the complete-wetting limit, $\cos\theta_e \simeq 1$, this reduces to

$$\Delta p_{\text{cap}} = \frac{\gamma(r-1)}{\varepsilon H} = \frac{\gamma}{\varepsilon}\frac{4W}{P^2}, \tag{S56}$$

which is independent of H, since both the wetted sidewall area and the invaded liquid volume scale linearly with the pillar height.

In one dimension, if this capillary pressure drop is applied over a wetted distance $L(t)$, then $p_x \sim -\Delta p_{\text{cap}}/L$ and $q \sim M_{\text{sat}}\Delta p_{\text{cap}}/L$. Using $\dot{L} = q/(\varepsilon H)$, one obtains

$$L^2(t) = 2\frac{M_{\text{sat}}}{\varepsilon H}\Delta p_{\text{cap}}\, t, \tag{S57}$$

recovering the Washburn-like diffusive scaling reported in hemiwicking experiments [5, 6].

For micropillar arrays with $H < P$, the wicking speed is expected to increase with pillar height, whereas for $H > P$ it should saturate and become governed mainly by the lateral spacing [7, 8]. The present mobility expressions capture both limits through the crossover at $H \sim w_{\text{eq}}$.

Figure S5 compares predicted and reported wicking diffusion coefficients for representative geometries from the literature. In Fig. S6, their ratio is plotted as a function of the confinement factor H/w_{eq}. The ratio scatter around 0.75 over the whole range of H/w_{eq} spanned, showing good consistency of the predicted mobility. The systematic deviation can be attributed to a limitation of the forcing model, in particular the simple thermodynamic estimate of Δp_{cap} and the scaling arguments, rather than to the mobility (which recovers the expected behaviors at $h < H$).

The predicted values are of the same order as the reported measurements and support the microchannel approximation as a reasonable estimate of the dominant viscous resistance governing hemiwicking.

S4.2 Application to vapor-mediated wetting control

The Marangoni mobility derived here can be used to interpret situations in which a surface-tension gradient opposes capillary imbibition. If a gradient of order $\gamma_x \sim -\Delta\gamma/L$ develops over the wetted film, the one-dimensional spreading law becomes

$$L^2(t) = 2\frac{1}{\varepsilon H}\left(M_{\text{sat}}\Delta p_{\text{cap}} - N_{\text{sat}}\Delta\gamma\right) t. \tag{S58}$$

This predicts slower, or even arrested, wicking when Marangoni forcing opposes capillary driving.

A complete description of that problem would require coupling the thin-film equation to vapor-phase transport and to concentration transport within the liquid, including Taylor-dispersion effects [9]. This lies beyond the scope of the present work. Nevertheless, the mobility framework developed here provides the essential hydrodynamic ingredient needed for such coupled models.

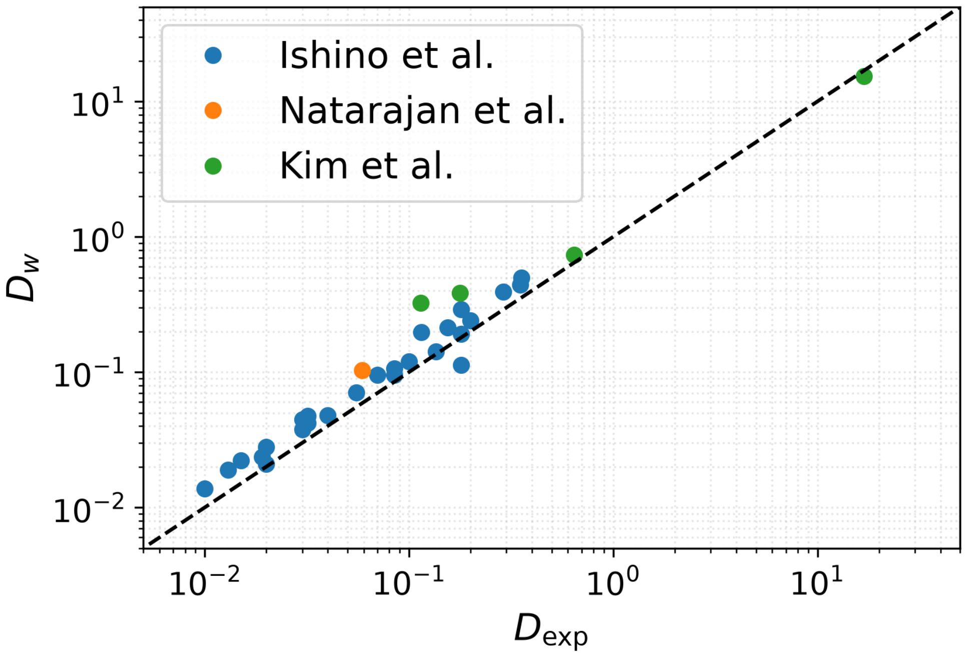

Fig. S5 Comparison of predicted and reported wicking diffusion coefficients. The prediction uses $D_w = 2M_{\mathrm{sat}}\Delta p_{\mathrm{cap}}/(\varepsilon H)$ for complete wetting. Data are taken from [8] for a cylindrical array of radius R, yielding $\varepsilon = 1 - \pi R^2/P^2$ and $\Delta p_{\mathrm{cap}} = \frac{\gamma}{\varepsilon}\frac{2\pi R}{P^2}$.

When Marangoni forcing balances capillary imbibition,

$$N_{\mathrm{sat}}\nabla\gamma \approx M_{\mathrm{sat}}\nabla p_{\mathrm{cap}}, \tag{S59}$$

the imbibed film may arrest at a finite extent, leading to coexisting droplet–film states as reported in Ref. [10]. Since $N/M \sim h^{-1}$, the critical thickness for this balance is naturally of order $h \sim \varepsilon P$ for geometries with $H \sim P$.

References

[1] Diddens, C., Rocha, D.: Bifurcation tracking on moving meshes and with consideration of azimuthal symmetry breaking instabilities. Journal of Computational Physics **518**, 113306 (2024)

[2] Heil, M., Hazel, A.L.: oomph-lib – an *O*bject-*O*riented *M*ulti-*Ph*ysics finite-element *Lib*rary. In: Fluid-structure Interaction: Modelling, Simulation, Optimisation, pp. 19–49. Springer, Berlin, Heidelberg (2006)

[3] Bauer, C., Frink, A., Kreckel, R.: Introduction to the ginac framework for symbolic computation within the c++ programming language. Journal of Symbolic

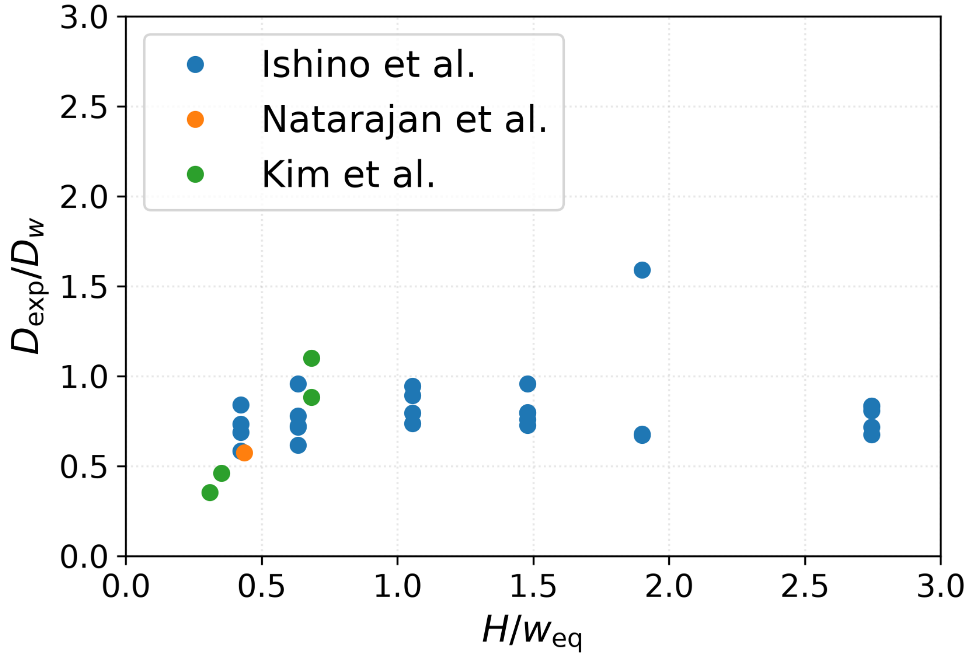

Fig. S6 Ratio of measured to predicted wicking diffusion coefficient, as a function of the confinement factor H/w_{eq}, for datasets from [8] for a cylindrical array of radius R, yielding $\varepsilon = 1 - \pi R^2/P^2$ and $\Delta p_{\text{cap}} = \frac{\gamma}{\varepsilon}\frac{2\pi R}{P^2}$.

Computation **33**(1), 1–12 (2002)

[4] Quéré, D.: Wetting and roughness. Annu. Rev. Mater. Res. **38**(1), 71–99 (2008)

[5] Bico, J., Tordeux, C., Quéré, D.: Rough wetting. Europhysics Letters **55**(2), 214 (2001)

[6] Kim, J., Moon, M.-W., Kim, H.-Y.: Dynamics of hemiwicking. Journal of Fluid Mechanics **800**, 57–71 (2016)

[7] Ishino, C., Reyssat, M., Reyssat, E., Okumura, K., Quere, D.: Wicking within forests of micropillars. Europhysics letters **79**(5), 56005 (2007)

[8] Natarajan, B., Jaishankar, A., King, M., Oktasendra, F., Avis, S.J., Konicek, A.R., Wadsworth, G., Jusufi, A., Kusumaatmaja, H., Yeganeh, M.S.: Predicting hemiwicking dynamics on textured substrates. Langmuir **37**(1), 188–195 (2020)

[9] Mekhitarian, L., Sobac, B., Dehaeck, S., Haut, B., Colinet, P.: Evaporation dynamics of completely wetting drops on geometrically textured surfaces. Europhysics Letters **120**(1), 16001 (2017)

[10] Xu, Z., Saiseau, R., Ramírez-Soto, O., Karpitschka, S.: Vapor-mediated wetting and imbibition control on micropatterned surfaces. Proceedings of the National Academy of Sciences **123**(1), 2519761122 (2026)
